# Genetic diversity, and description of a new dagger nematode, *Xiphinema afratakhtehnsis* sp. nov., (Dorylaimida: Longidoridae) in natural forests of southeastern Gorgan, northern Iran

**DOI:** 10.1371/journal.pone.0214147

**Published:** 2019-05-01

**Authors:** Zeinab Mirzaie Fouladvand, Ebrahim Pourjam, Pablo Castillo, Majid Pedram

**Affiliations:** 1 Department of Plant Pathology, Faculty of Agriculture, Tarbiat Modares University, Tehran, Iran; 2 Instituto de Agricultura Sostenible (IAS), Consejo Superior de Investigaciones Científicas (CSIC), Córdoba, Spain; Universidade de Coimbra, PORTUGAL

## Abstract

The most prevalent dagger nematode recovered from rhizospheric soil samples of forest trees in the Afrātakhteh region of Golestan province (Iran) was *Xiphinema afratakhtehnsis* sp. nov. and it is described and illustrated with integrative approaches using both morphological and molecular criteria. It belongs to the morphospecies group 6 of the intragenic historical grouping of *Xiphinema* non-*americanum* species. The new species is characterized by females with 3.3–4.9 mm sized body, lip region separated from the rest of body by a depression, anteriorly expanded, 16–18 μm wide, vulva located at 47.2–58.5%, odontostyle 155–173 μm and odontophore 89–107 μm long, female genital system composed of two equally developed branches, the tubular part of each having spines, short symmetrically rounded female tail to symmetrically rounded with a small mucro-like projection at the end in a few females, rare males (n = 1 out of 74 females) with 83 μm long dorylaimoid spicules and four juvenile developmental stages. The third-stage juveniles (J3) have a characteristic tail shape (short, symmetrically conical with a club-shaped long mucro) demarcating the species, and being typologically useful for its separation from closely similar species (except *X*. *cohni*, with currently no data on its juvenile stages) *viz*. *X*. *adenohystherum*, *X*. *iranicum*, *X*. *mazandaranense*, *X*. *nuragicum*, *X*. *pyrenaicum*, *X*. *robbinsi*, *X*. *sphaerocephalum* and *X*. *zagrosense*. Molecular phylogenetic studies using genomic (partial large subunit and internal transcribed spacer 1 ribosomal RNA genes: D2-D3 and ITS1 rDNA) and mitochondrial cytochrome c oxidase subunit I gene (*COI* mtDNA) revealed the new species forming a unique lineage in all reconstructed trees using Bayesian inference (BI) and maximum likelihood (ML) methods. The sequenced isolates of the new species formed a monophyletic group in the D2-D3 tree. The sequenced isolates of the new species for their *COI* mtDNA formed four subclades in *COI* mtDNA phylogeny, and four haplotypes in corresponding analysis.

## Introduction

The dagger nematode genus *Xiphinema* was established in 1913 by Cobb [1] with the monotypic designation of *X*. *americanum* Cobb, 1913 [1]. According to Andrássy [2], it is now not only the largest nematode genus in the order Dorylaimida Pearse, 1942 [3], but also the largest genus in the phylum Nematoda Potts, 1932 [4]. According to Loof and Luc [5], 172 nominal *Xiphinema* non-*americanum* species were described up until 1990. For pragmatic identification purposes, these authors separated the *Xiphinema* non-*americanum* group into several morphospecies groups (1 to 8), although they did not analyze phylogenetic relationships. Between 1990 and 1996, two supplements to the original identification key including 31 additional species were also published [6,7]. However, corrections to the characteristic features of some species listed in these keys were proposed after updated observations: *viz*. *X*. *vuittenezi* Luc, Lima, Weischer & Flegg 1964 [8] was transferred to the morphospecies group 6 after observation of spines in the uterus [9], *X*. *barense* Lamberti, Roca, Agostinelli and Bleve-Zacheo 1986 [10] was first transferred to the group 7 in the second supplement of the key [7] after confirmation of a hemispheroid rather than a conoid tail, and was later transferred to group 5 following observation of spines + pseudo Z in the uterus [11], *X*. *clavatum* Heyns, 1965 [12] was transferred from group 8 to group 5 after observation of a pseudo-Z organ in the uterus [13], *X*. *pyrenaicum* Dalmasso, 1969 [14] was transferred to group 6 following confirmation of spines in the uterus [7], and *X*. *paulistanum* Carvalho, 1965 [15] was transferred to group 2 [7]. Coomans et al. [16] and Andrássy [2] included 197 and 209 species under the genus in non-*americanum* group respectively. From 1996 (after publication of the second supplement key) to the present, 54 extra species were added to the genus and five previously synonymized species (their synonymization was accepted by Coomans et al. [16]) have been revalidated using molecular phylogenetic studies [17–55] (S1 Table).

*Xiphinema* spp. have a cosmopolitan distribution and according to Hunt [56], feed in the root tip zone to the hairy root region and aggregate at appropriate feeding sites. Darkening of the feeding point, lateral root proliferation and gall formation are other detected symptoms [57]. Currently, nine species of *Xiphinema* are proven vectors of plant pathogenic viruses (genus *Nepovirus*, family *Comoviridae*) [58,59]. However, the biodiversity and virus vectoring capacity of members of this genus might be underreported since many species have been described in the last 20 years without studies to detect their ability to transmit plant pathogenic viruses.

In the last decade, several species of *Xiphinema* native to Iran have been described *viz*. *X*. *robbinsi* Pedram, Niknam & Decraemer 2008 [46], *X*. *iranicum* Pedram, Niknam, Robbins, Ye & Karegar 2009 [31], *X*. *granatum* Pedram, Pourjam, Palomares-Rius, Ghaemi, Cantalapiedra-Navarrete & Castillo 2012 [27], *X*. *mazandaranense* Pedram, Pourjam, Robbins, Ye, Atighi & Decraemer 2012 [37], *X*. *zagrosense* Ghaemi, Pourjam, Pedram, Robbins, Ye & Decraemer 2012 [54] and *X*. *castilloi* Roshan-Bakhsh, Pourjam, Pedram, Robbins & Decraemer 2014 [23]; all of which were characterized with molecular data in their original description, except *X*. *robbinsi* which was analyzed by its molecular phylogenetic characters using topotype individuals [60]. From the abovementioned species, the species *X*. *mazandaranense* occurs in high densities in forests of Salaheddin Kola, Mazandaran province, in association with beech trees (*Fagus orientalis* Lipsky). However, no research on the occurrence of *Xiphinema* spp. in the forests of Golestan province has been conducted. Our surveys in September 2017 to identify *Xiphinema* species associated with forest trees in Golestan province, northern Iran, yielded several populations of a *Xiphinema* non-*americanum* group species, in most of the collected soil samples (24 out of 45 soil samples, the GPS information and the isolate codes in S2 Table) in natural forests, south and east of the city of Gorgan, Golestan province, northern Iran, that were all typologically similar (the occurrence point of sequenced populations are given in S1 Fig). Close morphological examination of all recovered populations and their phylogenetic analyses using several individuals/populations and different ribosomal and mitochondrial markers, revealed that all the populations were identical (especially in having the same tail morphology in the third-stage juvenile (J3), and close genetic relationships based on ribosomal and mitochondrial DNA markers). All morphological and molecular data indicated that these populations belong to an undescribed species of *Xiphinema* which is described and illustrated herein as *Xiphinema afratakhtehnsis* sp. nov.

The objectives of this research were to *i*) describe *Xiphinema afratakhtehnsis* sp. nov. based on several populations recovered from northern of Iran using morphological and molecular data, and *ii*) determine intraspecies genetic variation of the recovered populations and infer their molecular phylogenetic relationships with other *Xiphinema* non-*americanum* group species.

## Material and methods

### Ethics statement

No specific permits were required for the indicated field studies. The samples from forests were obtained in public areas, and do not involve any species endangered or protected in Iran, nor are the sites protected in any way.

#### Sampling, nematode extraction, mounting and drawing

Soil samples were collected from southeastern natural forests of the city of Gorgan, Golestan province, northern Iran, during September 2017. The samples were collected at random from several locations, east of Gorgan. The nematodes were extracted from soil using a series of 20, 60 and 270 mesh sieves (USA standard mesh numbers) having 850, 250, 53 μm openings size, respectively. The individuals of interest were handpicked under a Nikon SMZ1000 stereomicroscope, each recovered individuals from each soil sample was regarded as an independent population. The recovered specimens were heat-killed by adding boiling 4% formalin solution, transferred to anhydrous glycerin according to De Grisse´s method [61]. For studying the uterine structure, live females were selected, killed by gentle heat in a drop of water, studied and photographed in temporary slides. The permanent slides were examined using a Nikon Eclipse E600 light microscope. Photographs were taken using an Olympus DP72 digital camera attached to an Olympus BX51 microscope equipped with differential interference contrast (DIC) optics. Drawings were made using a drawing tube attached to the microscope and were redrawn using CorelDRAW software version X6. The morphometric study of each nematode population included classic diagnostic features in Longidoridae (i.e. de Man body ratios, lip region and amphid shape, oral aperture-guiding ring, odontostyle and odontophore length) [62]. All measurements were expressed in micrometers (μm), unless otherwise indicated in the text. The juvenile stages were identified according to Robbins et al. [63]. All abbreviations used are as defined in Jairajpuri & Ahmad [62]. For comparisons with closely related species, their original descriptions were checked. For some species, updated characters, e.g. new observations on uterus differentiation types and juvenile characters, were also used. Voucher specimens of this described species have been deposited in the nematode collection of Faculty of Agriculture, Tarbiat Modares University, Tehran, Iran and USDA Nematode Collection, Beltsville, MD.

Two Google Earth 5.1 (Google, Mountain View, United States of America) and ArcGIS 9.3 (ESRI, 2009, 2013) software programs were used to schematically map the GPS data of the sequenced populations points (S1 Fig).

#### DNA extraction, PCR, sequencing, and phylogenetic analyses

For molecular analyses, three to six females from each population were collected (the code of isolates are given in S2 Table), mounted in water and their uterus observed and photographed. Then the nematodes were transferred to a clean slide into 10 μl TE buffer, crushed with a coverslip, and the solution was collected by adding an additional 50 μl TE buffer (10 mM Tris-Cl, 0.5 mM EDTA; pH 9.0, Qiagen). Each of these samples was regarded as an independent DNA sample (each DNA extraction was from a single nematde). DNA samples were stored at −20° C until used as template for PCR. Primers for 18S rDNA amplification were forward primer F22 (5′-TCCAAGGAAGGCAGCAGGC-3′) [64] and reverse primer 18S 1573R (5ʹ-TACAAAGGGCAGGGACGTAAT-3’) [65]. Primers for D2-D3 amplification were forward primer D2A (5′-ACAAGTACCGTGAGG GAAAGT-3′) and reverse primer D3B (5′-TGCGAAGGAACCAGCTACTA -3′) [66] and the reverse primer KK28S-4 (5-ʹGCGGTATTTGCTACTACCAYYAMGATCTGC-3ʹ) [67]. The ITS1 region was amplified using forward primer rDNA1 (5′-TTGATTACGTCCCTGCCCTTT-3′) and reverse primer rDNA1.58S (5′-ACGAGCCGAGTGATCCACCG- 3′) [68], and finally, the cytochrome c oxidase subunit 1 gene (*COI*) was amplified using a combination of the below primers: forward primer COIF (5′-CATTTTTTGGKCATCCWGARG-3ʹ) and reverse primer XIPHR2 (5ʹ-GTACATAATGAAAATGTGCCAC-3ʹ) [69] and forward primer JB3 (5ʹ-TTTTTTGGGCATCCTGAGGTTTAT-3ʹ) [70]. The thermal cycling program for amplification of all aforementioned genomic and non-genomic fragments was as follows: denaturation at 95°C for 3–6 min, followed by 35 cycles of denaturation at 94°C for 30 s, annealing at 52–55°C (except for *COI*, 45–48°C) for 30–60 s, and extension at 72°C for 1 min. A final extension was performed at 72°C for 10 min. DNA sequencings were performed using the same primers used in PCR. All the newly generated sequences were deposited into the GenBank database under the accession numbers presented in S2 Table.

The newly obtained sequences (18S, D2-D3, ITS1 rDNA and *COI*) were compared with those of other *Xiphinema* species available in GenBank using the BLAST homology search program. For reconstructing the phylogenies (18S and *COI* phylogenies), we used sequences from the last phylogenetic study [44] with minor changes (by adding newly generated and some other sequences from the database) and for ITS1 phylogeny, the updated sequences of Archidona-Yuste *et al*. [20] were used. For D2-D3 phylogeny, the maximal number of species were also included. The sequences were aligned using the Q-INS-i algorithm of the online version of MAFFT v. 7 (http://mafft.cbrc.jp/alignment/server/) [71]. The Gblocks program (version 0.91b) with all the three less stringent parameters (http://phylogeny.lirmm.fr/phylo_cgi/one_task.cgi?task_type=gblocks), was used for post-editing of the alignments. The model of base substitution for each dataset was selected using MrModeltest 2 [72]. Bayesian analysis was performed using MrBayes v3.1.2 [73] running the chains for 10× 10^6^ (18S, ITS and *COI*) and 5×10^6^ generations (D2-D3). After discarding burn-in samples, the remaining samples were retained for further analyses. The Markov chain Monte Carlo (MCMC) method within a Bayesian framework was used to estimate the posterior probabilities of the phylogenetic trees [74] using the 50% majority rule. Convergence of model parameters and topology were assessed based on average standard deviation of split frequencies and potential scale reduction factor values. Adequacy of the posterior sample size was evaluated using autocorrelation statistics as implemented in Tracer v.1.6 [75]. A maximum likelihood (ML) tree was reconstructed by RaxmlGUI 1.1 [76] software using the same nucleotide substitution model as in the BI in 1000 bootstrap (BS) replicates for all four datasets. The output files of the phylogenetic programs (MrBayes and RaxmlGUI 1.1) were visualized using Dendroscope V.3.2.8 [77] and were re-drawn in CorelDRAW software version 16. The Bayesian posterior probability (BPP) and ML BS values exceeding 0.50 and 50%, respectively, are given on appropriate clades in the shape of BPP/ML BS.

#### Haplotype networking

The haplotype frequencies were estimated using DNASP version 6.0 [78] and the NETWORK 5 software [79] was used to infer a median joining (MJ) network among mtDNA haplotypes.

#### Nomenclatural acts

The electronic edition of this article conforms to the requirements of the amended International Code of Zoological Nomenclature (ICZN), and hence the new name contained herein is available under that Code from the electronic edition. This published work and the nomenclatural acts it contains have been registered in ZooBank, the online registration system for the ICZN. The ZooBank LSIDs (Life Science Identifiers) can be resolved and the associated information viewed through any standard web browser by appending the LSID to the prefix "http://zoobank.org/". The LSID for this publication is: urn:lsid:zoobank.org:pub:44164C94-F309-4288-82C2-A8A153ECC04F. The electronic edition of this work was published in a journal with an ISSN, and has been archived and is available from the following digital repositories: PubMed Central, LOCKSS.

## Results

### *Xiphinema afratakhtehnsis* mirzaei fouladvand, Pourjam, Castillo and Pedram sp. nov.

urn:lsid:zoobank.org:act:9955F617-37FD-4912-8D95-9EC6DD1BF868

**Fig 1:**
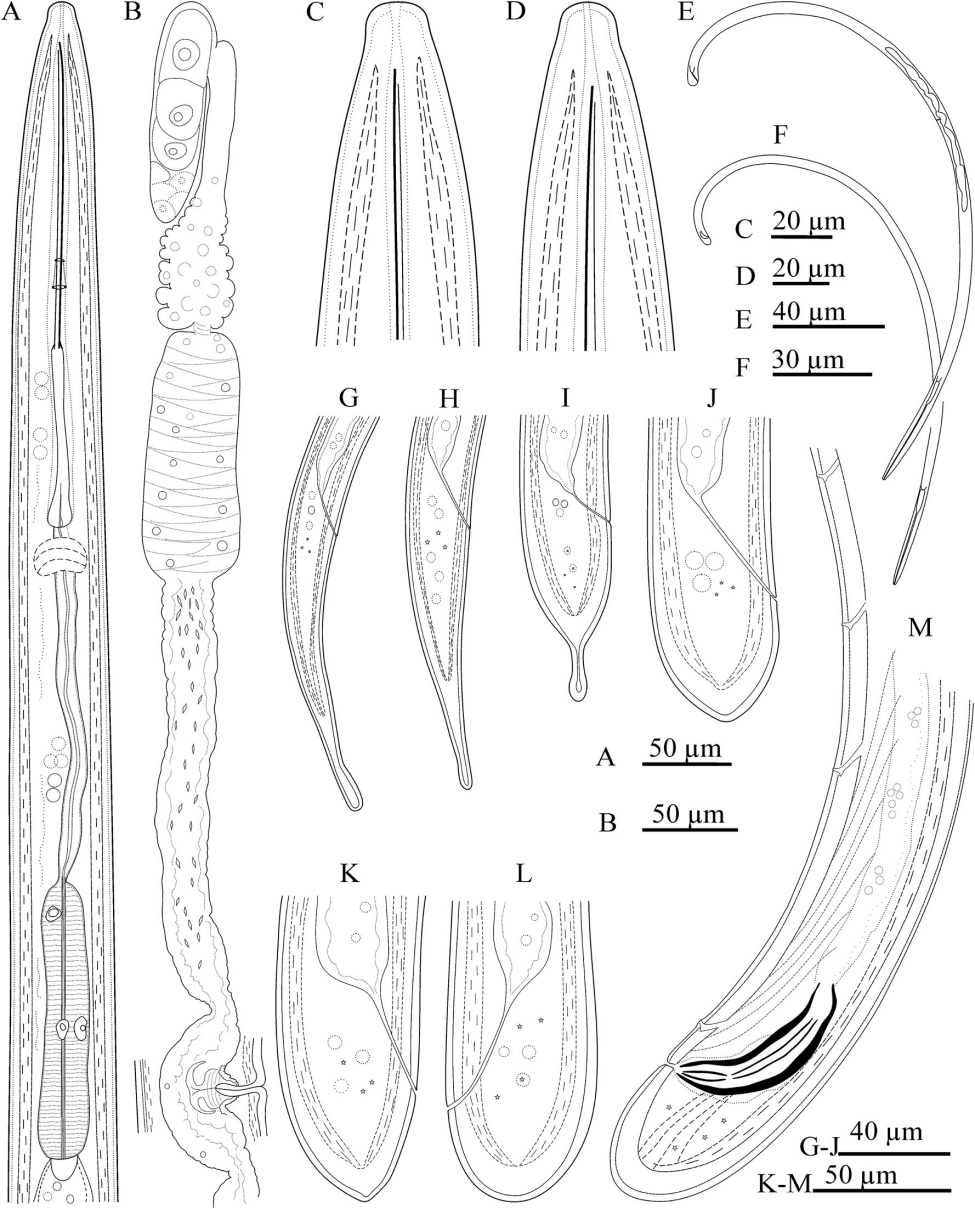
Line drawings of *Xiphinema afratakhtehnsis* sp. nov. (female holotype and paratype juveniles). A) Pharyngeal region. B) Female anterior genital branch. C-D) Male and female lip region. E-F) Female and male entire body. G-J) Tail of juvenile developmental stages (J1-J4) respectively. K&L) Female tail. M) Male tail.

**Fig 2:**
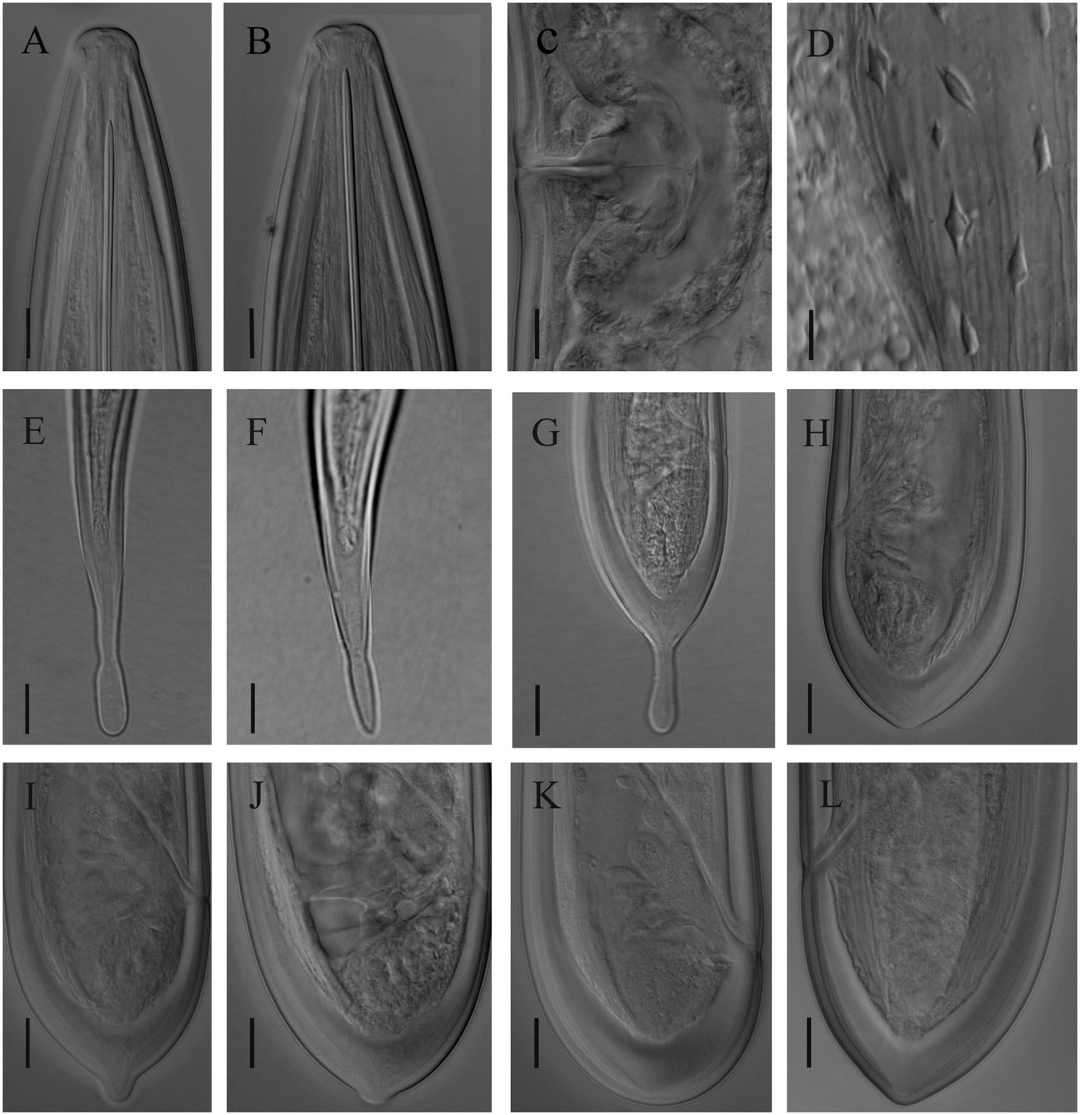
Light micrographs of *Xiphinema afratakhtehnsis* sp. nov., female paratypes and and J1-J4 stages. A&B) Anterior region. C) Vulval region. D) Spines in uterus of fresh females. E-H) Tail of juvenile developmental stages (J1-J4) respectively. I&J) Variation in J4 tail. K&L) Female tail shape (K: the common shape, L: tail end with bulge observed in few females). Scale bars = 10 μm.

**Fig 3:**
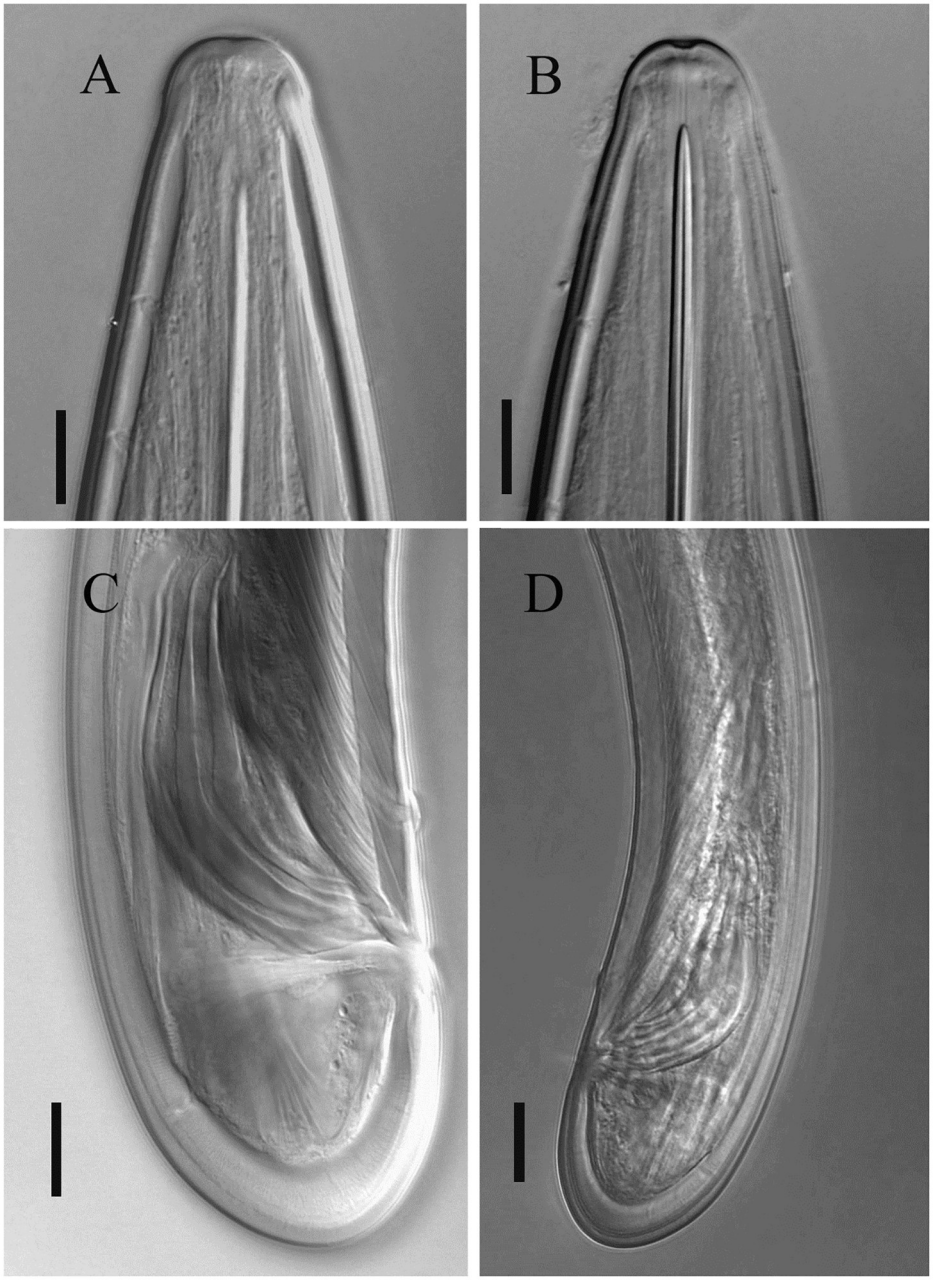
Light micrographs of *Xiphinema afratakhtehnsis* sp. nov. (A, C, D) and *X*. *mazandaranense* (B). A) Typical lip region of the new species, B) Typical lip region of *X*. *mazandaranense*. C&D) Tail, spicule and posterior body region of the male of the new species. Scale bars: A, B, C = 10 μm, D = 20 μm).

Figs 1, 2, 3A, 3C and 3D

#### Holotype

Adult female, collected from the rhizosphere of common bracken (*Pteridium aquilinum* (L.) Kuhn) at Afrātakhteh, in eastern Gorgan; mounted in pure glycerin and deposited in the nematode collection at Nematode Collection of the Faculty of Agriculture, Tarbiat Modares University, Tehran, Iran (collection number TM5010).

#### Paratypes

Adults and juvenile paratypes extracted from soil samples collected from the same locality as the holotype; mounted in pure glycerin and deposited in the following nematode collections: five paratype females, two paratype juveniles from each stage and populations of other localities deposited at the Nematode Collection of the Faculty of Agriculture, Tarbiat Modares University, Tehran, Iran (collection numbers TM5011-TM5036); five paratype females and two paratype juveniles from each stage were deposited at USDA Nematode Collection, Beltsville, MD; eight paratype females and other paratype juveniles at WANECO collection, Wageningen, The Netherlands (http://www.waneco.eu/); and several voucher specimens at Ghent University Museum, Zoology Collections, Ghent, Belgium, Nematode Collection of the Faculty of Agriculture, Tarbiat Modares University, Tehran, Iran and USDA Nematode Collection, Beltsville, MD.

#### Diagnosis

*Xiphinema afratakhtehnsis* sp. nov. is a parthenogenetic species characterized by medium sized females 3.3–4.9 mm long, anteriorly wide-expanded lip region 16–18 μm wide, 155–173 μm long odontostyle, 89–107 μm long odontophore, vulva located at 47.2–58.5%, two equally developed female genital tracts having discernible spines in fresh and permanent mounts, symmetrically rounded short tail sometimes with a small bulge at the end in few females, rare male (one male out of 74 females), and four juvenile developmental stages, J1-J4 (representing the first to fourth stage respectively), with J3 having a characteristic tail morphology, helping discriminate it from eight out of nine morphologically close species. It belongs to the morphospecies group 6 sensu Loof and Luc [5] and the polytomous identification codes of the new species are as follows (not common forms inside parentheses): A4, B3, C7(5), D56, E56, F345, G34, H2, I3, J5, K2, L1.

#### Etymology

The species name refers to the type locality of the type population of the new species (code 801), Afrātakhteh, east to southeast of the city of Gorgan.

#### Description of taxa. Female

Body cylindrical, ventrally curved after fixation, more at posterior region, forming an open J, very gradually narrowing toward anterior end. Cuticle smooth under light microscopy, comprising two layers, the fine striae visible on outer layer mostly in tail, 4.5–6.0 μm thick in anterior region, varying to 5.5–7.0 μm at mid body and 7.0–8.5 μm in anus, the hyaline part of tail 13–16 μm thick. Lateral chord 15–18 μm wide, occupying 20.3–26.5% of corresponding body diam. Lip region anteriorly smooth, expanded, separated from the rest of the body by a shallow depression, 1.8–2.0 times wider than high. Amphidial fovea cup-shaped; aperture a wide slit at slightly anterior to cephalic region-body junction. Odontostyle typical of the genus, long and slender, 7.5–9.2 times the lip region diameter or 1.5–1.7 times the odontophore length. Odontophore with well-developed flanges. Guiding ring double. Pharynx consisting of an anterior slender narrow part extending to a terminal pharyngeal bulb with three gland nuclei. The larger dorsal gland nucleus (DN) at 12.3–15.9% of pharyngeal bulb length, the two smaller ventrosublateral nuclei (S1N) at about the same level, at 52.0–59.2% of pharyngeal bulb length (location of gland nuclei according to Loof & Coomans [80]). Cardia conical, 15–18 × 16–20 μm in size. Intestine simple, prerectum 7.7–12.0 times and rectum 0.6–0.9 times anal body width long. Female reproductive system didelphic-amphidelphic, the branches about equally developed, each composed of a 90–127 μm long ovary, a 113–157 μm long oviduct with well-developed *pars dilatata oviductus*, a sphincter and a 370–565 μm long bipartite uterus composed of *pars dilatata uteri* and a tubular part with 4–5 μm long spines well discernible in fresh individuals and permanently mounted specimens, well developed ovejector, vagina perpendicular to body axis, 47.4–59.7% of corresponding body width long and vulva a transverse slit. Tail symmetrically rounded, with a small bulge at the end in few females.

#### Male

Rare (only one male recovered out of 74 females). General morphology similar to that of female, except for characters related with sexuality. Genital system diorchic, testes opposed. Spicules paired, massive, 5.7 times longer than wide, lateral accessory pieces 20 μm long. Copulatory supplements composed of a cloacal pair, and three single ventromedian supplements, the distal one at 233 μm distance (hiatus) from the cloacal pair. Tail similar to that of female, symmetrically rounded.

#### Juveniles

**Fig 4:**
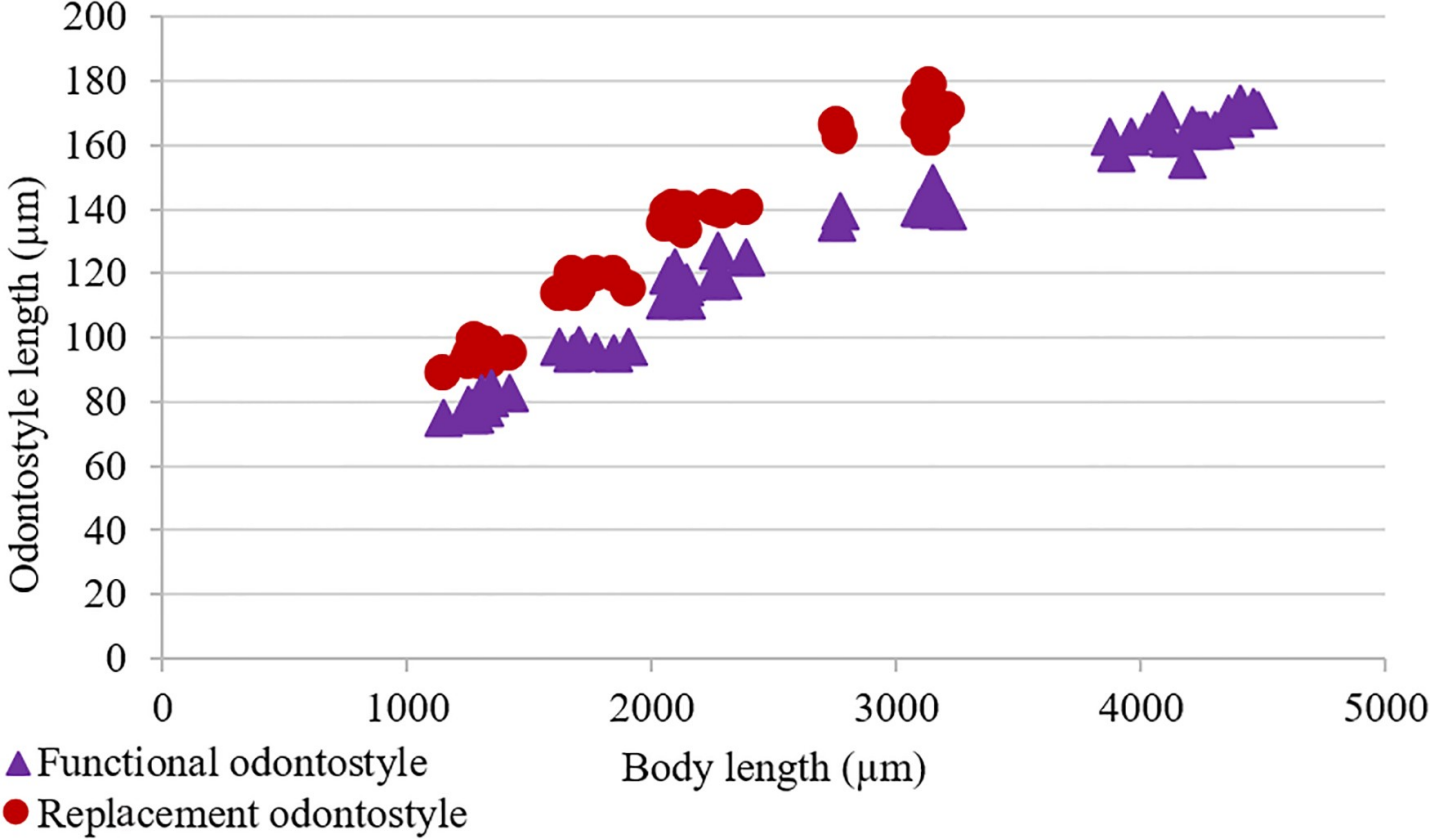
Relationship between body length and functional and replacement odontostyle length in all developmental stages from first-stage juveniles (J1) to mature females of *Xiphinema afratakhtehnsis* sp. nov. (type population, code: 801).

All four juvenile developmental stages were identified for the type population (code 801, see Table 1) according to Robbins et al. [63]. For the population code 764, three latter juvenile stages were recovered (see Table 2). The correlation between body length, replacement and functional odontostyle of the type population is given in Fig 4. Lip region in all juvenile stages looks similar to that in females. J1 is characterized by the replacement odontostyle tip close to the base of the functional odontostyle, and is located at the level with the odontophore. In all other stages, the replacement odontostyle is posterior to the flanges of odontophore in its resting position. J1 has an elongate conoid, ventrally slightly bent tail, with a wide digit-like offset mucro at the end, J2 has an elongate conoid tail with a less offset narrower (compared to that in J1) mucro at the end, J3 has a symmetrically conical tail, convex on both sides, ending in a long club-like extension, and J4 has a short conical tail, convex on both sides, ending in a small bulge.

**Table 1:** Morphometrics of the type population of *Xiphinema afratakhtehnsis* sp. nov. **(population code 801)**^a^.

Character/Ratio^b^	J1*	J2*	J3*	J4*	Female	
	Paratypes	Paratypes	Paratypes	Paratypes	Paratypes	Holotype
n	11	7	11	9	18	1
L	1293.4±67.7	1743.6±102.3	2168.4±108.1	3059.3±172.1	4191.8±187.9	4210
	(1150.0–1417.5)	(1617.5–1905.0)	(2057.5–2390.0)	(2760.0–3212.5)	(3867.5–4477.5)	
a	46.9±3.8	44.7±2.3	48.4±5.0	47.6±3.3	56.8±4.8	62.8
	(40.3–53.5)	(40.3–47.3)	(41.9–56.8)	(43.7–54.2)	(50.9–66.8)	
b	3.7±0.2	4.2±0.4	4.3±0.1	4.9±0.3	6.3±0.3	6.6
	(3.4–4.0)	(3.9–5.2)	(4.1–4.6)	(4.5–5.3)	(5.7–6.8)	
c	12.5±0.7	19.9±1.1	32.8±1.8	58.9±4.4	86.7±7.3	95.7
	(11.0–13.6)	(18.6–22.0)	(30.6–35.3)	(54.2–66.9)	(75.9–97.9)	
c'	5.5±0.5	3.5±0.2	1.9±0.1	1.0±0.1	0.8±0.1	0.8
	(4.3–6.0)	(3.0–3.7)	(1.7–2.1)	(0.9–1.1)	(0.7–1.0)	
V	-	-	-	-	51.0±2.2	49.8
					(47.2–55.1)	
Lip height	5.0±0.2	6±0	6.3±0.3	7±0	9±1	9
	(5.0–5.5)	(6–6)	(6.0–6.5)	(7–7)	(8–10)	
Lip width	10.2±0.3	11.4±0.5	12.5±0.4	14±0	16.6±0.7	17
	(10–11)	(11–12)	(12–13)	(14–14)	(16–18)	
Odontostyle length	79.5±3.1	96.3±1.1	117.3±4.1	141.0±3.4	165.7±4.6	166
	(75–84)	(95–98)	(112–125)	(136–148)	(157–173)	
Odontophore length	48.9±3.6	60.6±1.4	73.1±2.7	88.7±2.7	102.0±3.4	102
	(39–53)	(59–63)	(69–77)	(85–93)	(96–107)	
Stylet total length	128.4±3.4	156.9±1.3	190.4±4.1	229.7±4.7	267.7±6.0	268
	(123–135)	(155–159)	(183–196)	(223–238)	(259–277)	
Replacement odontostyle	93.9±2.9	116.9±3.0	138.5±3.0	168.0±5.8	-	-
	(89–99)	(114–120)	(133–141)	(162–179)		
Anterior end to vulva	-	-	-	-	2136.0±82.6	2097.5
					(2000.0–2287.5)	
Anterior end to guiding ring	67.9±4.3	91.4±1.6	110.0±2.4	136.6±5.2	163.2±7.2	158
	(59–74)	(90–94)	(107–115)	(126–143)	(153–176)	
Flange width	-	-	-	-	14.8±1.8	12
					(12–19)	
Pharynx length	348.0±22.8	416.5±28.0	501.6±17.7	620.6±24.0	668.9±26.7	640
	(317.5–407.5)	(368–460)	(475.0–527.5)	(590.0–667.5)	(615–713)	
Pharyngeal expansion length	83.5±5.6	95.6±7.3	116.5±5.2	135.9±4.8	154.8±6.2	157
	(71–89)	(86–105)	(111–125)	(127–142)	(145–165)	
Pharyngeal expansion diam.	15.4±1.0	19.4±2.0	22.7±2.1	25.3±2.4	30.3±2.2	28
	(14.0–16.5)	(16–22)	(20–26)	(22–29)	(28–35)	
Body width at mid body	27.7±2.1	39.1±3.1	45.2±4.6	64.4±4.8	74.1±3.9	67
	(24–31)	(36–44)	(37–50)	(57–71)	(67–79)	
- at anus	18.9±1.9	25.2±1.9	34.6±3.0	51.3±3.9	58.8±3.9	55
	(17–24)	(23–28)	(30–39)	(47–56)	(54–67)	
- at guiding ring level	23.5±1.1	30.2±3.6	35.8±3.6	47.8±2.9	54.3±3.8	49
	(22–25)	(27.5–38.0)	(30–43)	(43.0–50.5)	(48–60)	
Prerectum length	-	-	-	-	569.9±52.3	555
					(507.5–682.5)	
Rectum length	-	-	-	-	52.6±4.9	55
					(46.5–65.5)	
Hyaline tail region	37.4±3.9	40.9±3.7	31.2±2.3	13.8±1.7	14.7±1.1	15
	(31–43)	(36–45)	(27.5–35.0)	(12–17)	(13–16)	
Tail	103.9±2.2	87.6±5.3	66.2±3.2	52.1±3.7	48.6±3.2	44
	(100–106)	(83–95)	(60–70)	(48–58)	(44–53)	

*J1-J4 refers to first to fourth juvenile developmental stages respectively.

(-) Not obtained or not performed.

^a^ Measurements are in μm and in the form: mean ± standard deviation (range).

^b^ Abbreviations as defined in Jairajpuri & Ahmad [62]. a, body length/maximum body width; b, body length/pharyngeal length; c, body length/tail length; c', tail length/body width at anus; V (distance from anterior end to vulva/body length) x 100.

**Table 2:** Morphometrics of *Xiphinema afratakhtehnsis* sp. nov. (population with code 764)^a^.

Character/Ratio^b^	J2*	J3*	J4*	Female
n	1	7	16	20
L	1667.5	2241.5±114.8	2946.6±243.9	4126.3±327.2
		(2061.3–2350.0)	(2420.0–3242.5)	(3722.5–4885.0)
a	50.5	51.3±3.3	56.8±3.4	63.3±6.7
		(47.0–55.4)	(50.4–62.3)	(51.5–74.3)
b	4.3	4.7±0.5	5.2±0.5	6.9±0.7
		(4.1–5.4)	(4.2–6.0)	(6.1–8.5)
c	23.5	37.9±4.1	62.1±5.0	92.4±8.4
		(34.7–46.1)	(48.4–68.5)	(74.5–106.9)
c'	3.1	1.8±0.3	1.1±0.1	0.9±0.1
		(1.3–2.0)	(1.0–1.3)	(0.7–1.1)
V	-	-	-	51.0±2.7
				(48.8–58.5)
Lip height	6	6.3±0.3	7±0	9.0±0.8
		(6.0–6.5)	(7–7)	(8–10)
Lip width	12	12.7±0.4	14±0	17.0±0.8
		(12–13)	(14–14)	(16–18)
Odontostyle length	88	114.8±4.4	139.6±7.2	165.7±4.6
		(110–121)	(120–149)	(155–172)
Odontophore length	59	75.7±2.9	84.7±6.2	96.5±4.5
		(70–78)	(67–94)	(89–103)
Stylet total length	147	190.5±6.4	224.4±12.6	262.2±9.1
		(180–198)	(187–236)	(244–275)
Replacement odontostyle	110	140.5±4.8	162.7±5.5	-
		(132–145)	(147–172)	
Anterior end to vulva	-	-	-	2102.9±163.7
				(1877.5–2456.3)
Anterior end to guiding ring	76	103.0±2.2	128.1±9.6	149.2±8.5
		(100–106)	(98–140)	(135–166)
Flange width	**-**	-	-	14.2±1.0
				(13.0–15.5)
Pharynx length	385.5	476.7±47.7	564.6±22.1	595.0±23.1
		(412.5–550.0)	(520.0–610.4)	(545.0–632.5)
Pharyngeal expansion length	84	109.5±3.3	127.8±6.1	137.4±8.1
		(106–115)	(116–139)	(122–149)
Pharyngeal expansion diam.	28	41.0±2.9	47.3±3.7	33.1±2.9
		(37–45)	(43–55)	(27–37)
Body width at mid body	33	43.8±3.3	51.9±2.9	65.7±6.5
		(41–50)	(46–57)	(56–78)
- at anus	23	34.2±3.2	43.2±1.9	52.5±4.5
		(31–40)	(40–47)	(44–61)
- at guiding ring level	23	34.8±3.2	39.2±2.4	47.0±3.5
		(30–40)	(36–43)	(41–57)
Prerectum length	-	-	-	627.4±113.8
				(400–880)
Rectum length	-	-	-	47.0±6.3
				(35–55)
Hyaline tail region	20	23.3±4.7	13.2±2.4	14.7±1.5
		(14–26)	(11–18)	(12–17)
Tail	71	59.7±5.9	47.5±3.3	45.0±5.2
		(51–67)	(42–54)	(38–61)

*J1-J4 refers to first to fourth juvenile developmental stages respectively.

(-) Not obtained or not performed.

^a^ Measurements are in μm and in the form: mean ± standard deviation (range).

^b^ Abbreviations as defined in Jairajpuri & Ahmad [62]. a, body length/maximum body width; b, body length/pharyngeal length; c, body length/tail length; c', tail length/body width at anus; V (distance from anterior end to vulva/body length) x 100.

#### Measurements, morphology and distribution

Morphometric variability is described in Tables 1 and 2 and S3 Table and morphological traits shown in Figs 1, 2 and 3A, 3C and 3D. In addition to the type locality, *Xiphinema afratakhtehnsis* sp. nov. was collected from the rhizosphere of *Rubus* sp., *Quercus* sp. and *Hedera helix* L., all of which were located in two localities of Golestan and Semnan provinces, their GPS information, host plant of several other populations as well as the isolates codes and accession numbers are given in S2 Table.

#### Relationships

By having two equally developed female genital branches, short-rounded tail of females and presences of spines in tabular part of the uterus, *Xiphinema afratakhtehnsis* sp. nov. morphologically resembles nine known *Xiphinema* species namely: *X*. *adenohystherum* Lamberti, Castillo, Gomez-Barcina & Agostinelli 1992 [18], *X*. *cohni* Lamberti, Castillo, Gomez-Barcina & Agostinelli 1992 [18], *X*. *iranicum*, *X*. *mazandaranense*, *X*. *nuragicum* Lamberti, Castillo, Gomez-Barcina & Agostinelli 1992 [18], *X*. *pyrenaicum*, *X*. *robbinsi*, *X*. *sphaerocephalum* Lamberti, Castillo, Gomez-Barcina & Agostinelli 1992 [18] and *X*. *zagrosense*. Compared with all aforementioned species, except *X*. *cohni* with currently no data on its juvenile stages, the new species has a diagnostic til shape of J3. The extensive comparisons with the abovementioned species are included below:

Compared with *X*. *adenohystherum*, by distant placement in all four phylogenetic trees, wider lip region (16–18 *vs* 10.0–12.9 μm), smaller c (74.5–106.9 *vs* 126.8–149.6), longer odontostyle (155–173 *vs* 143.3–151.8 μm) and odontophore (89–107 *vs* 79.4–88.2 μm) and longer tail (38–61 *vs* 29.4–35.3 μm).

Compared with *X*. *cohni*, by distant placement in all four phylogenetic trees, wider lip region (16–18 *vs* 14.7–15.9 μm) and its tail shape (rounded and rounded with a small mucro at terminus *vs* widely conoid with widely rounded terminus).

Compared with *X*. *iranicum*, by distant placement in ITS1 tree, slightly greater ć (0.7–1.1 *vs* 0.5–0.8), smaller c ratio (74.5–106.9 *vs* 119.5–154.3), longer tail (38–61 *vs* 26–41 μm) and differently shaped tail (symmetrically rounded to symmetrically rounded with a small mucro *vs* rounded, dorsally more convex usually with a minute terminal mucro).

Compared with *X*. *mazandaranense*, by distant placement in 18S tree, wider lip region (16–18 *vs* 13–15 μm) anteriorly expanded lip region (*vs* rounded, see Fig 3A & 3B) and longer tail (38–61, average = 46 *vs* 33–44, average = 39.5 μm).

Compared with *X*. *nuragicum*, by distant placement in all four phylogenetic trees, longer odontophore (89–107 *vs* 77.1–87.1 μm) and longer tail (38–61 *vs* 32.3–42.2 μm).

Compared with *X*. *pyrenaicum*, by distant placement in all four phylogenetic trees, smaller b (5.3–8.5 *vs* 7.1–11.1) and c (74.5–106.9 *vs* 90–126) values, longer odontostyle (155–173 *vs* 127–149 μm), odontophore (89–107 *vs* 76–90 μm) and tail (38–61 *vs* 34–41 μm).

Compared with *X*. *robbinsi*, by its separate clade in 28S tree, lacking abundant males (*vs* having common functional males), longer body (3.3–4.9 *vs* 3.0–3.6 mm), longer odontostyle (155–173 *vs* 107.5–127.0 μm) and odontophore (89–107 *vs* 62.5–74.4 μm) and longer (38–61 *vs* 30.5–38.0 μm) and differently shaped tail (rounded and rounded with a minute bulge at terminus *vs* short conoid, dorsally convex, with widely rounded mucro at the end).

Compared with *X*. *sphaerocephalum*, by distant placement in all four phylogenetic trees, slightly longer tail (38–61, average = 46 *vs* 32.4–44.7, average = 35.9 μm) and basic differences in tail characters of J1 (longer (100–106 *vs* 58–59 μm), elongate conoid, ventrally bent, with a wide digit-like offset mucro at the end *vs* conical, dorsally slightly convex, ventrally slightly concave with a wide rounded tip).

Compared with *X*. *zagrosense*, by distant placement in both 18S and 28S trees, and basic difference in tail shape of J1-J3 as follows: a ventrally bent elongate conoid tail, with a wide digit-like offset mucro at the end in J1, 100–106 μm long *vs* a dorsally convex conoid tail with a 15 μm long cuticular extension in shape of a digitate mucro, 67–80 μm long, elongate conoid tail with a less offset narrower mucro at the end (compared to that in J1) *vs* a dorsally convex conoid tail with a cuticular extension in J2, symmetrically conical, convex on both sides, ending in a long club-like extension *vs* conical with a rounded end with a subdigitate extension in J3.

#### Molecular divergence of the new species

The PCR amplification of D2-D3 segments, ITS1 region, the partial 18S rDNA and partial *COI* mtDNA regions yielded single fragments of *ca* 900, 1100, 1800 and 500 bp, respectively, based on gel electrophoresis. Sequences from *X*. *afratakhtehnsis* sp. nov. matched well with the *X*. non-*americanum* group species sequences deposited in GenBank, being clearly different from all of them. Twelve new D2-D3 of 28S rDNA sequences from *X*. *afratakhtehnsis* sp. nov. were obtained in the present study (MH429073-MH429078, MH429080-MH429085). The D2-D3 segments of *X*. *afratakhtehnsis* sp. nov. showed 97% nucleotide similarity (differing by 23 to 27 nucleotides and 2 to 4 indels) with *Xiphinema granatum* (JQ240273). Intraspecific variability among the 12 studied populations was low, only 11 variable positions were found in D2-D3. ITS1 sequences of *X*. *afratakhtehnsis* sp. nov. (MH429086-MH429097) matched closely with the *X*. non-*americanum* group spp. deposited in GenBank, *X*. *aceri* (EU477385) being the most related species for this region, which showed a similarity value of 91% (differing by 81 nucleotides and 16 indels). As well as in the D2-D3 of the 28S rDNA, the intraspecific variability found for the 12 populations studied was low, showing only 10 variable positions. The partial 18S rDNA from *X*. *afratakhtehnsis* sp. nov. showed a high nucleotide similarity with the 18S rDNA sequences from *X*. non-*americanum* group species, being 99% similar to all of the accessions from this group deposited in GenBank. Finally, 16 *COI* sequences from *X*. *afratakhtehnsis* sp. nov. were obtained in this study (MH429098-MH429113). The BLAST search using these new *COI* sequences, revealed the maximum identity was for *X*. *pyrenaicum* (KY816644), being 81% similar (differing by 73 to 76 nucleotides and no indels). In contrast to other regions studied in this work, the intraspecific variability found in the 16 *COI* sequences included was high, the pairwise distance of these 16 sequences ranged from 0.00 to 16.3%. (from 0 to 54 nucleotides) (S4 Table).

#### Phylogenetic relationships of *Xiphinema afratakhtehnsis* sp. nov. within *Xiphinema*

**Fig 5:**
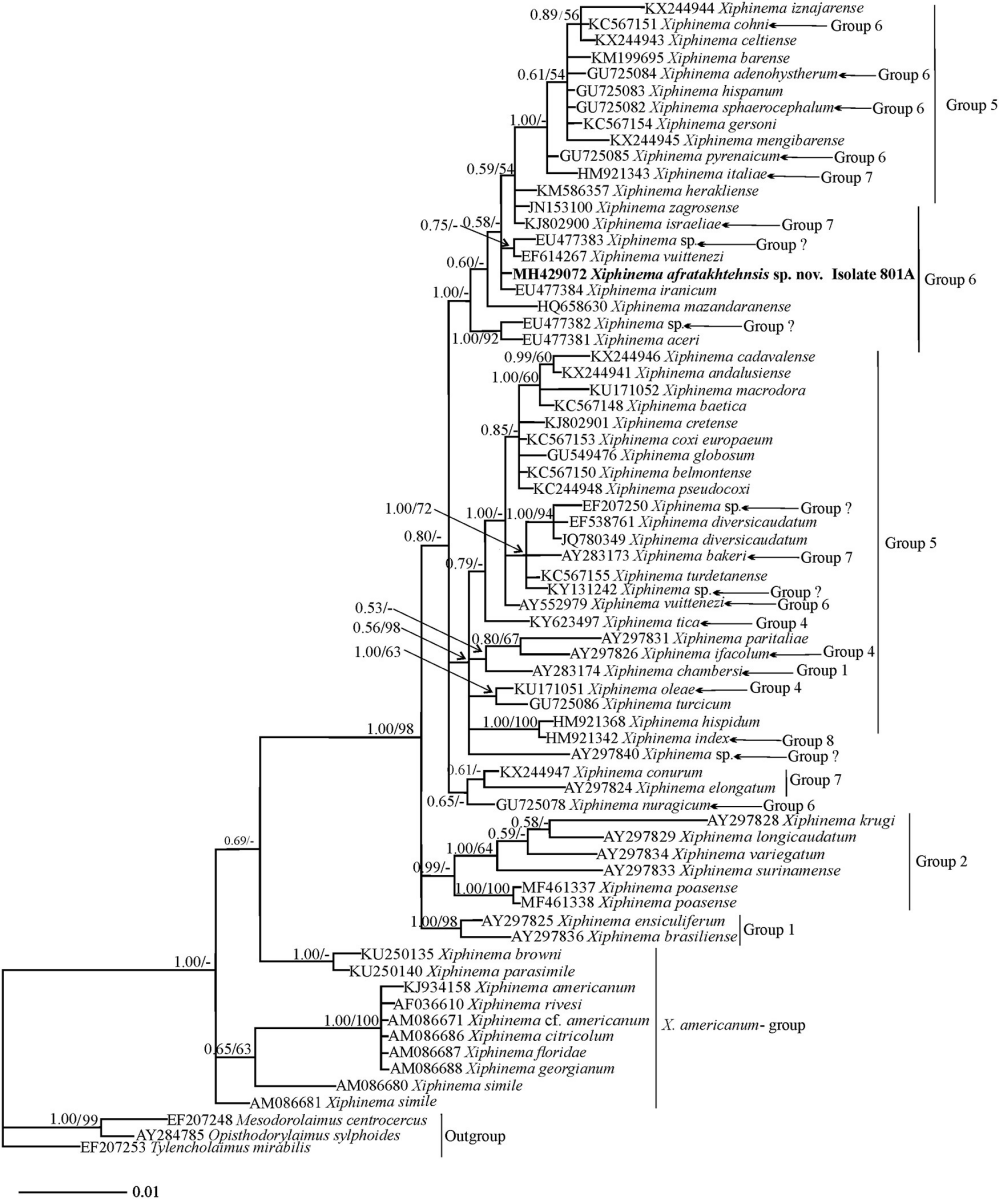
The 50% majority rule consensus trees from Bayesian inference analysis generated from the partial 18S rDNA dataset of *Xiphinema afratakhtehnsis* sp. nov. with the GTR+I+G model. Bayesian posterior probabilities and maximum likelihood bootstrap values are given for appropriate clades in the shape BPP/ML BS. Newly obtained sequences are in bold letters. Scale bar = expected changes per site.

**Fig 6:**
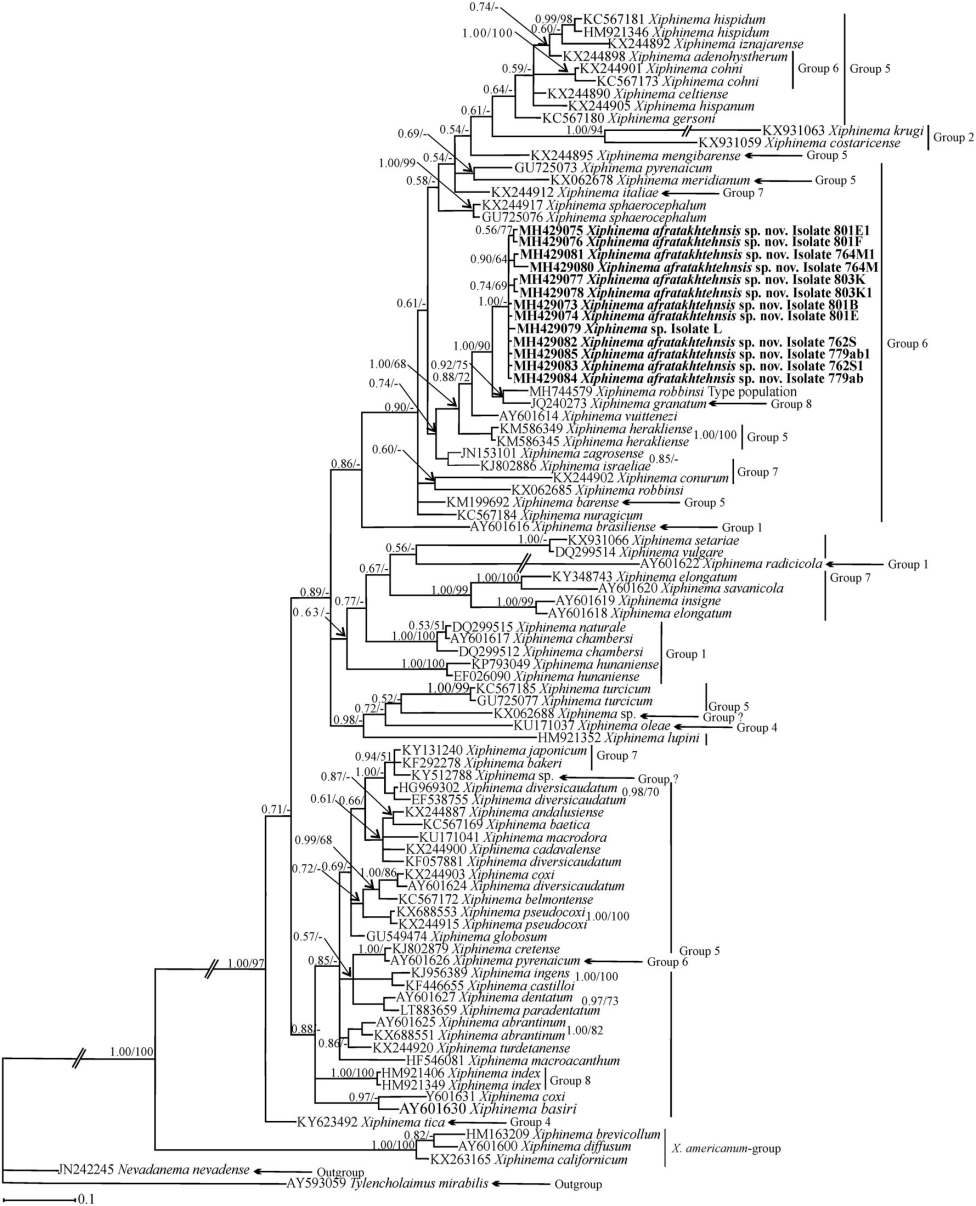
The 50% majority rule consensus trees from Bayesian inference analysis generated from the partial 28S rDNA dataset of *Xiphinema afratakhtehnsis* sp. nov. with the GTR+I+G model. Bayesian posterior probabilities and maximum likelihood bootstrap values are given for appropriate clades in the shape BPP/ML BS. Newly obtained sequences are in bold letters. Scale bar = expected changes per site.

**Fig 7:**
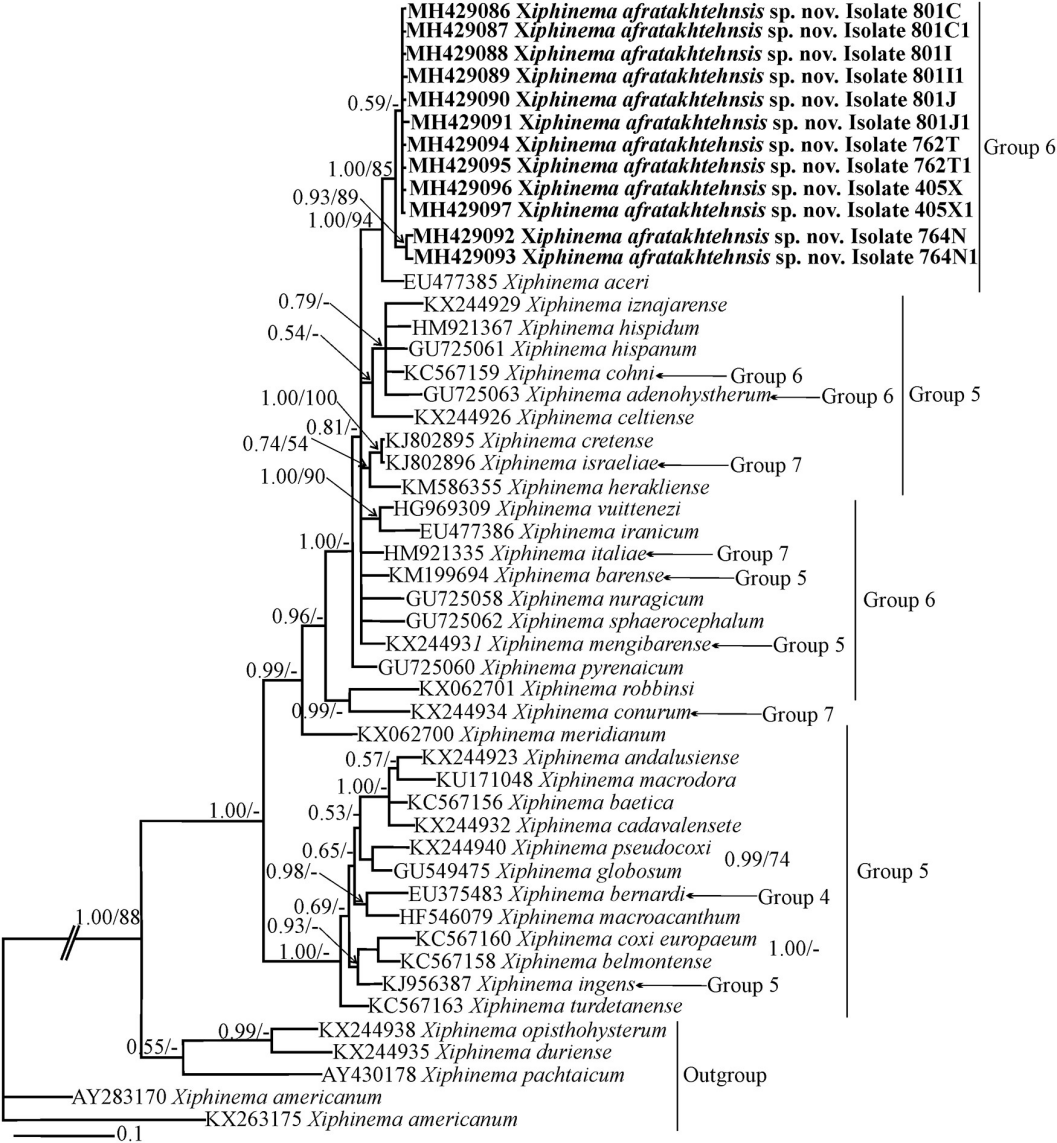
The 50% majority rule consensus trees from Bayesian inference analysis generated from the partial ITS1 rDNA dataset of *Xiphinema afratakhtehnsis* sp. nov. with the GTR+I+G model. Bayesian posterior probabilities and maximum likelihood bootstrap values are given for appropriate clades in the shape BPP/ML BS. Newly obtained sequences are in bold letters. Scale bar = expected changes per site.

**Fig 8:**
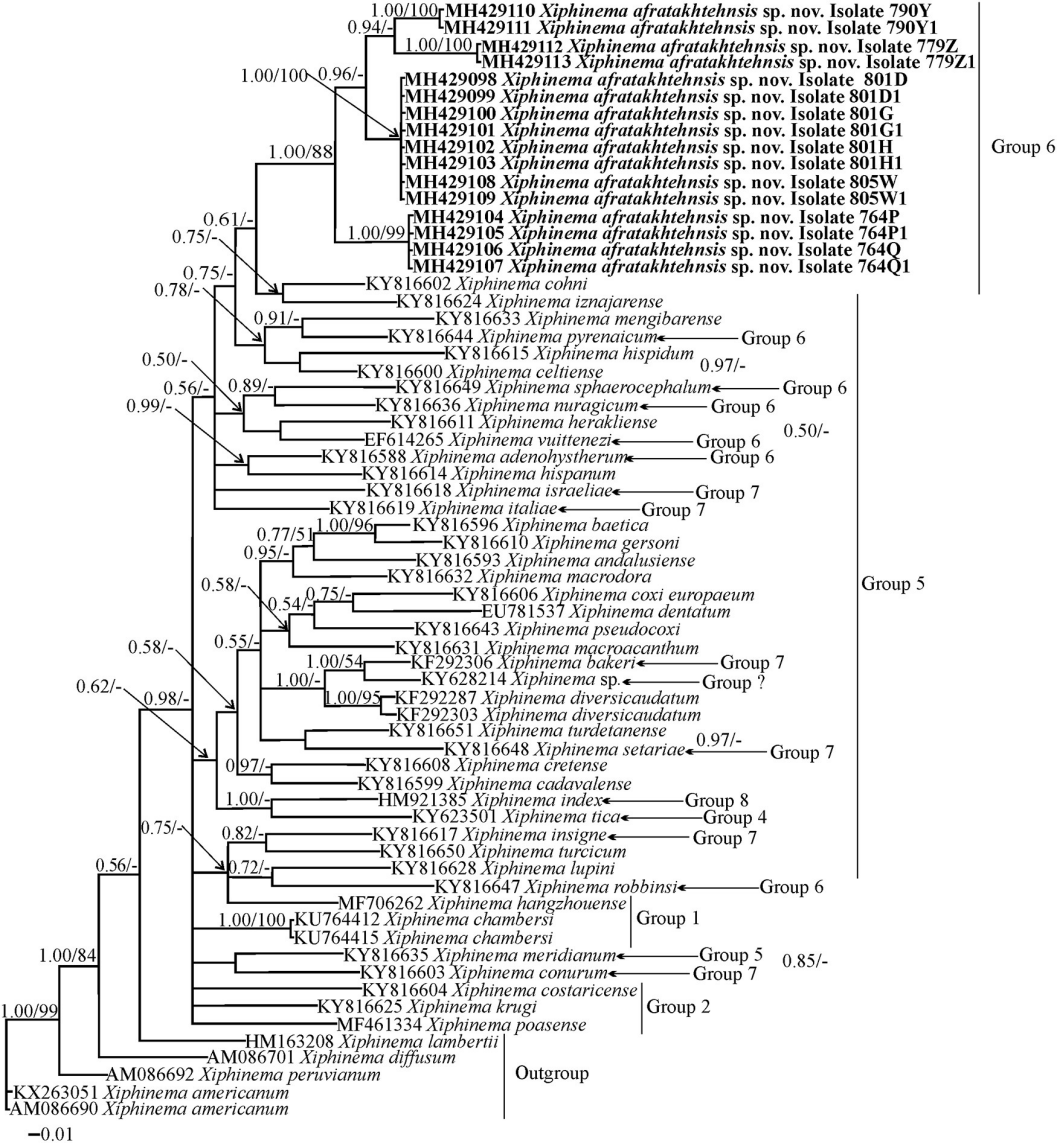
The 50% majority rule consensus trees from Bayesian inference analysis generated from the partial *COI* mtDNA gene dataset of *Xiphinema afratakhtehnsis* sp. nov. with the GTR+I+G model. Bayesian posterior probabilities and maximum likelihood bootstrap values are given for appropriate clades in the shape BPP/ML BS. Newly obtained sequences are in bold letters. Scale bar = expected changes per site.

Phylogenetic relationships among *X*. non-*americanum* group species inferred from analyses of partial 18S, D2-D3 and the ITS1 rDNA and *COI* gene sequences using BI and ML are given in Figs 5, 6, 7 and 8 respectively. The phylogenetic relationships using the partial 18S region for *X*. *afratakhtehnsis* sp. nov. (Fig 5) showed several clades but they were not well resolved. In this tree, *Xiphinema afratakhtehnsis* sp. nov. clustered with other species from the morphospecies group 6 (*X*. *vuittenezi*, EF614267; *X*. *iranicum*, EU477384; and one unidentified isolate of *Xiphinema* sp., EU477383), however this clade did not show well supported values (Fig 5). The D2-D3 tree (Fig 6) based on a multiple edited alignment of 95 sequences with 676 total characters revealed two major, but not well-supported clades, *X*. *afratakhtehnsis* sp. nov. appeared in the superior clade forming a well-supported subclade with *X*. *granatum* (JQ240273) and *X*. *robbinsi* (MH744579). An unidentified *Xiphinema* sp. (MH429079) recovered only as one single juvenile and sequenced in this study showed 99% similarity with *X*. *afratakhtehnsis* sp. nov. (differing by 3 to 7 bp and no indels) appeared to also belong to this species (Fig 6), but needs confirmation with additional material. For partial ITS1 rDNA and *COI* genes (Figs 7 and 8), the 50% majority-rule BI and ML trees of a multiple sequence alignment (55 sequences and 1019 characters and 47 sequences and 367 characters, respectively) showed a superior well supported clade formed by *X*. *afratakhtehnsis* sp. nov. accessions. This clade seems to be related to *X*. *aceri* for the ITS1 since both species clustered within a well-supported clade. In contrast, *X*. *afratakhtehnsis* sp. nov. did not form a well supported clade with other *Xiphinema* non-*americanum* species included in the *COI* tree.

The species *X*. *robbinsi* [46] that was originally described based on traditional criteria, was reported in Tunisia and molecularly characterized by 28S, ITS1 and *COI* with the accession numbers KX062685, KX062701 and KY816647, respectively Guesmi-Mzoughi et al. [81]. This Tunisian population has some morphological differences from the type population and the exact status of this population might need further confirmation (based on recent unpublished molecular studies in Iran).

#### Haplotype networking

**Fig 9:**
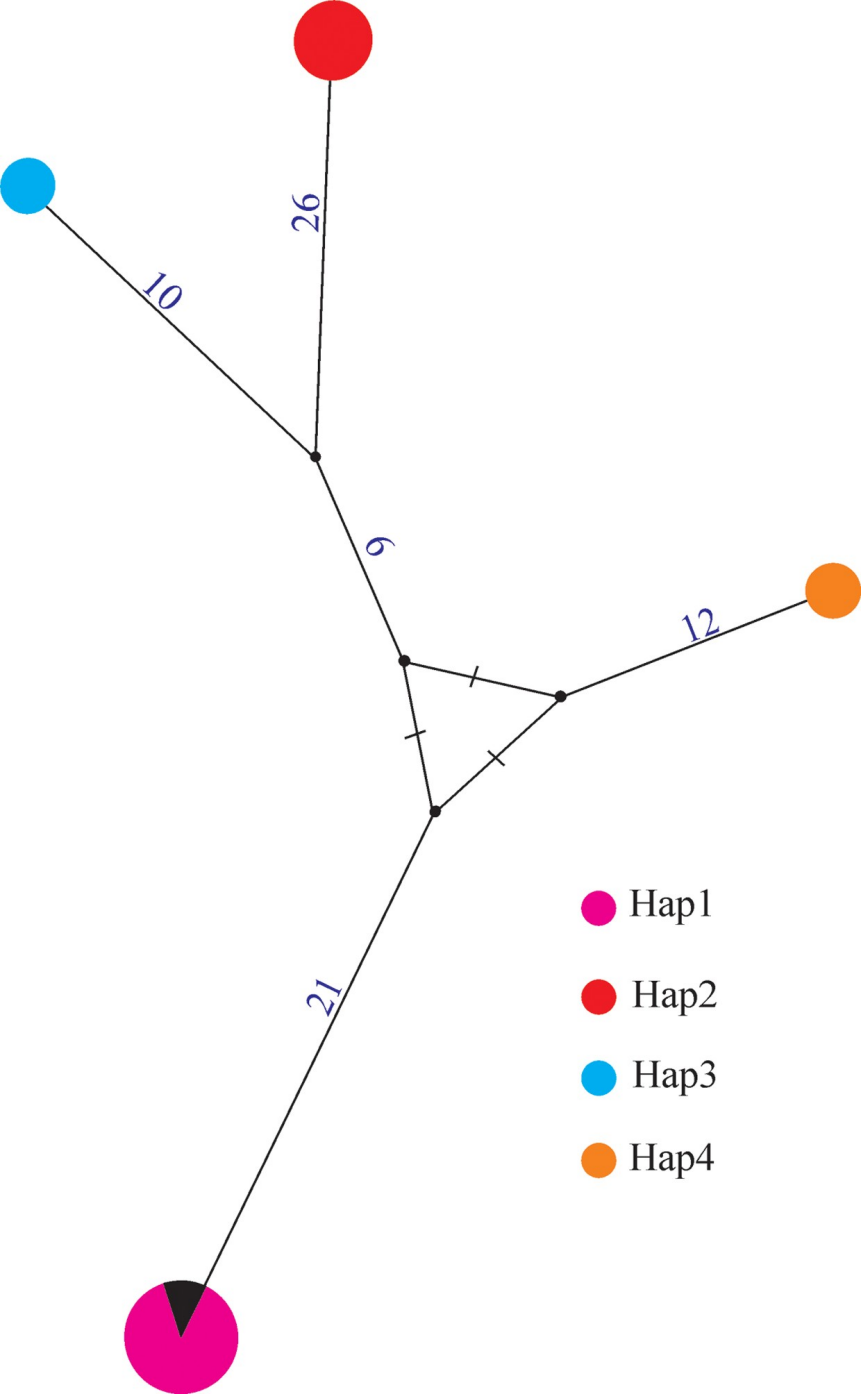
Minimum spanning network showing the relationships between haplotypes of five populations of *Xiphinema afratakhtehnsis* sp. nov. using *COI* mtDNA. Small black cycles represent missing haplotypes and the mutational steps are given on the branches.

The 16 sequenced specimens of five populations of *X*. *afratakhtehnsis* sp. nov. for *COI* mtDNA (S1 Fig) were separated into 4 haplotypes that were coded as Hap1 to Hap4 (Fig 9, S5 Table). The haplotypes Hap1, Hap3, and Hap4 included females with the shared shape of the tail tip (symmetrically rounded), while females have a small bulge at the tail tip belong to Hap2. Hap1 haplotype included individuals of the population codes 801 and 805. Hap2 included the population with code 764, Hap3 included the population with code 790, and Hap4 included the population with code 779. The resolved network’s pattern was similar to the resolved relationships of the same isolates in the *COI* phylogenetic tree (Fig 8).

## Discussion

*Xiphinema afratakhtehnsis* sp. nov. was recovered in most soil samples collected from natural forests in southeastern Gorgan in this study. It has spines in uterus, a short rounded tail in females, a male independent reproduction mode, four juvenile developmental stages and a unique tail shape for J3. This is the first comprehensive study on the occurrence of *Xiphinema* in Golestan province, and the second time that a species of this genus was found to be highly distributed in natural forests of Iran. Formerly, *X*. *mazandaranense* was found highly distributed in forests of Salaheddin Kola, Mazandaran province [37]. The undisturbed nature of these forests and probably, the host preference of both species on natural forest trees could be the reason for such observations, and, this exposes a research area for evaluating the putative economic effects of these species on forest plants. The presently described new species has a rounded tail, and in few individuals had a small bulge-like projection at the tail tip. The third-stage juvenile has a unique shape of tail, facilitating its separation from several other closely related species. According to Coomans et al. [16] the tail shape in juveniles of *X*. non-*americanum* group is not common, and thus, could be a reliable morphological feature to primary delimit the species. The use of genomic rDNA markers has already been extensively used in species identification and phylogeny of longidorid nematodes, and except for the 18S, a slowly evolving marker that usually fails to resolve species [19,82,83], reliable species identifications using D2-D3 and ITS1 markers have been confirmed [21,23,24,37,60]. In our phylogenetic analyses, the new species appeared as an independent lineage, confirming the usefulness of these markers in taxonomic studies based on integrative approaches. In the case of the new species, the intraspecies variations were observed for D2-D3 and *COI* sequences, and cladogenesis events were observed in corresponding phylogenies, being more robust in *COI* tree. However, no remarkable intra-species variation was observed for ITS sequences of several populations; and as already discussed, the two separate accession numbers in our ITS tree had shorter lengths. In general, this marker could be useful as a suitable marker for species separation purposes, but difficulties persist with alignment [84].

Our phylogenetic analyses using ribosomal and mitochondrial markers, revealed the higher number of cladogenesis events happen using *COI* marker, separating the populations of the species to four sub-clades in the corresponding phylogeny, in accordance with the results of the network analyses. This marker has already been proven as a useful tool in taxonomy of the *Xiphinema americanum*-group members [85,86] and has been recently used for population analyses and haplotype networking studies of *Longidorus orientalis* Loof, 1982 [87,88] and *Longidorus poessneckensis* Altherr, 1974 [89,90]. In the latter study, a 17.1% intraspecies variation was observed for this marker, and a comparison with intraspecies variation was performed with some other species. As already given, the intraspecies variation of *COI* mtDNA for the new species ranged between 0.00–16.3%. There were however no remarkable morphological/morphometric differences between the populations sequenced for their *COI* mtDNA, and between the populations formed different clades in *COI* tree and the haplotype network.

Similar to a previous study using the *COI* marker for the description of *X*. *japonicum* Zhao, Ye, Maria, Pedram & Gu, 2017 [32], some polytomies were observed in our inferred *COI* tree. With regard to cladogenesis events in the new species, and overall congruence with the inferred haplotype analysis, the marker is well suitable for population study and intraspecies genetic variation inspection purposes. In the present haplotype analysis, five populations of the new species were sequenced for their *COI* mtDNA, resulted in four haplotype groups. Further sequencings of extra individuals from other populations could yield new haplotypes. And finally, the two accession numbers AY55297 and AY601626, were shown as *Xiphinema* sp. in D2-D3 and 18S trees in our phylogenies, due to uncertainty of their identity.

## Conclusions

In summary, this study provides new insights into the diversity and prevalence of the genus *Xiphinema* associated with forests in Iran, with the description of a new species (*Xiphinema afratakhtehnsis* sp. nov.) enlarging the diversity of this genus in the country.

## Supporting information

S1 Fig Geographic locations of sample sites (upper map) of which the recovered populations of the new species were sequenced for their genomin or non-genomic regions (this map was generated during present study, and may be similar but not identical to other published maps of Iran and is used only for the purpose of showing the abovementioned sites). The enlarged part shows Golestan province and the aforementioned points.
(TIF)

S1 Table *Xiphinema* Cobb, 1913 [1] species described after publication of the original identification key by Loof and Luc [5] and its supplements [6, 7], and the species revalidated after their synonymization. (DOCX)

S2 Table Information of the newly generated sequences in this study. (DOCX)

S3 Table Morphometrics of several populations of *Xiphinema afratakhtehnsis* sp. nov^a^. (DOCX)

S4 Table Pairwise distances between *COI* sequences of different isolates of *Xiphinema afratakhtehnsis* sp. nov^a^. (DOCX)

S5 Table Information of *COI* sequences of *Xiphinema afratakhtehnsis* sp. nov. used in haplotype analysis. (DOCX)
