# Supplementary figures and images for: Genetic diversity, and description of a new dagger nematode, Xiphinema afratakhtehnsis sp. nov., (Dorylaimida: Longidoridae) in natural forests of southeastern Gorgan, northern Iran
Source: PLoS One. 2019 May 1;14(5):e0214147. doi: 10.1371/journal.pone.0214147 (PMC6493718; doi:10.1371/journal.pone.0214147)

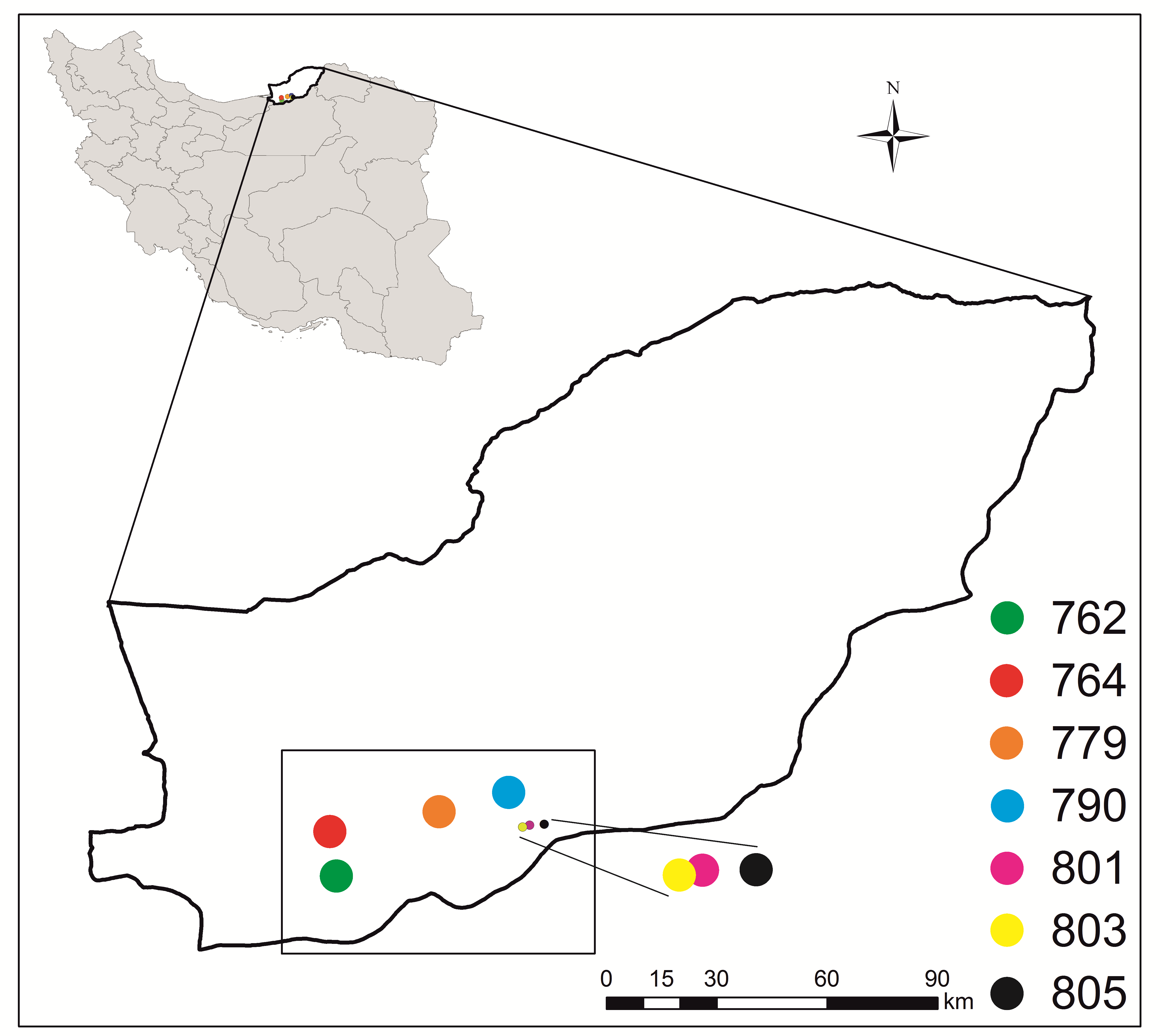

Supplement: S1 Fig — The enlarged part shows Golestan province and the aforementioned points. (TIF) [file pone.0214147.s001.tif]
