# Supplementary material for: Genetic diversity, and description of a new dagger nematode, Xiphinema afratakhtehnsis sp. nov., (Dorylaimida: Longidoridae) in natural forests of southeastern Gorgan, northern Iran
Source: PLoS One. 2019 May 1;14(5):e0214147. doi: 10.1371/journal.pone.0214147 (PMC6493718; doi:10.1371/journal.pone.0214147)
Supplement: S1 Table — (DOCX) [file pone.0214147.s002.docx]

**S1 Table**.

| **Species** | **Reference (original description)** | **Morphospecies group *sensu* Loof and Luc [5]** | **Reference (revalidated)** |
| --- | --- | --- | --- |
| 1. *Xiphinema aaba* | Heyns, 2000 [17] | 5 | Heyns [17] |
| 1. *Xiphinema adenohystherum* (R) | Lamberti, Castillo, Gomez-Barcina & Agostinelli, 1992 [18] | 6 | Gutiérrez‐Gutiérrez et al. [19] |
| 1. *Xiphinema andalusiense* | Archidona-Yuste, Navas-Cortes, Cantalapiedra-Navarrete, Palomares-Rius & Castillo, 2016 [20] | 5 | Archidona-Yuste et al. [20] |
| 1. *Xiphinema baetica* | Gutiérrez-Gutiérrez, Cantalapiedra-Navarrete, Remesal, Palomares-Rius, Navas-Cortes & Castillo, 2013 [21] | 5 | Gutiérrez-Gutiérrez et al. [21] |
| 1. *Xiphinema bernardi* | Robbins, Bae, Ye & Pedram, 2009 [22] | 4 | Robbins et al. [22] |
| 1. *Xiphinema castilloi* | Roshan-Bakhsh, Pourjam, Pedram, Robbins & Decraemer, 2014 [23] | 5 | Roshan-Bakhsh et al. [23] |
| 1. *Xiphinema celtiense* | Archidona-Yuste, Navas-Cortes, Cantalapiedra-Navarrete, Palomares-Rius & Castillo, 2016 [20] | 5 | Archidona-Yuste et al. [20] |
| 1. *Xiphinema cohni* (R) | Lamberti, Castillo, Gomez-Barcina & Agostinelli, 1992 [18] | 6 | Archidona-Yuste et al. [20] |
| 1. *Xiphinema cretense* | Tzortzakakis, Archidona-Yuste, Cantalapiedra-Navarrete, Nasiou, Lazanaki, Kabourakis, Palomares-Rius & Castillo, 2014 [24] | 5 | Tzortzakakis et al. [24] |
| 1. *Xiphinema densispinatum* | Barsi, Lamberti & Agostinelli, 1998 [25] | 7 | Barsi et al. [25] |
| 1. *Xiphinema enigmatum* | Siddiqi, 2000 [26] | 2 | Siddiqi [26] |
| 1. *Xiphinema granatum* | Pedram, Pourjam, Palomares-Rius, Ghaemi, Cantalapiedra-Navarrete & Castillo, 2012a [27] | 8 | Pedram et al. [27] |
| 1. *Xiphinema hangzhouense* | Cai, Maria, Barsalote, Subbotin & Zheng, 2018 [28] | 1 | Cai et al. [28] |
| 1. *Xiphinema herakliense* | Tzortzakakis, Archidona-Yuste, Cantalapiedra-Navarrete, Nasiou, Palomares-Rius & Castillo, 2015 [29] | 5 | Tzortzakakis et al. [29] |
| 1. *Xiphinema hispanum* (R) | Lamberti, Castillo, Gomez-Barcina & Agostinelli, 1992 [18] | 5 | Gutiérrez‐Gutiérrez et al. [19] |
| 1. *Xiphinema horvatovicae* | Barsi & Lamberti, 1999 [30] | 6 | Barsi & Lamberti [30] |
| 1. *Xiphinema illyricum* | Barsi & Lamberti, 1999 [30] | 6 | Barsi & Lamberti [30] |
| 1. *Xiphinema iranicum* | Pedram, Niknam, Robbins, Ye & Karegar, 2009 [31] | 6 | Pedram et al. [31] |
| 1. *Xiphinema iznajarense* | Archidona-Yuste, Navas-Cortes, Cantalapiedra-Navarrete, Palomares-Rius & Castillo, 2016 [20] | 5 | Archidona-Yuste et al. [20] |
| 1. *Xiphinema japonicum* | Zhao, Ye, Maria, Pedram & Gu, 2017 [32] | 7 | Zhao et al. [32] |
| 1. *Xiphinema kikuyense* | Heyns & Coomans, 1997 [33] | 5 | Heyns & Coomans [33] |
| 1. *Xiphinema labiosum** | Swart & Quénéhervé, 1998 [34] | 2 | Swart & Quénéhervé [34] |
| 1. *Xiphinema larliani* | Singh & Khan, 1997 [35] | 3 | Singh & Khan [35] |
| 1. *Xiphinema macedonicum* | Barsi & Lamberti, 1999 [30] | 6 | Barsi & Lamberti [30] |
| 1. *Xiphinema macrodora* | Archidona-Yuste, Navas-Cortes, Cantalapiedra-Navarrete, Palomares-Rius & Castillo, 2016 [20] | 5 | Archidona-Yuste et al. [20] |
| 1. *Xiphinema manasiae* | Sen, Chatterjee & Manna, 2010 [36] | 3 | Sen et al. [36] |
| 1. *Xiphinema masaiorum* | Heyns & Coomans, 1997 [33] | 5 | Heyns & Coomans [33] |
| 1. *Xiphinema mazandaranense* | Pedram, Pourjam, Robbins, Ye, Atighi & Decraemer, 2012 [37] | 6 | Pedram et al. [37] |
| 1. *Xiphinema mengibarense* | Archidona-Yuste, Navas-Cortes, Cantalapiedra-Navarrete, Palomares-Rius & Castillo, 2016 [20] | 5 | Archidona-Yuste et al. [20] |
| 1. *Xiphinema montenegrinum* | Barsi, Lamberti & Agostinelli, 1998 [25] | 5 | Barsi et al. [25] |
| 1. *Xiphinema mounporti* | Faye, Barsi & Decraemer, 2012 [38] | 3 | Faye et al. [38] |
| 1. *Xiphinema mwanzianum* | Coomans & Heyns, 1997 [39] | 5 | Coomans & Heyns [39] |
| 1. *Xiphinema naturale* | Lamberti, De Luca, Molinari, Duncan, Agostinelli, Coiro, Dunn & Radicci, 2002 [40] | 1 | Lamberti et al. [40] |
| 1. *Xiphinema neoradicicola* | Dhanam & Jairajpuri, 1997 [41] | 1 | Dhanam & Jairajpuri [41] |
| 1. *Xiphinema nuragicum* (R) | Lamberti, Castillo, Gómez-Barcina & Agostinelli, 1992 [18] | 6 | Gutiérrez‐Gutiérrez et al. [19] |
| 1. *Xiphinema oleae* | Archidona-Yuste, Navas-Cortes, Cantalapiedra-Navarrete, Palomares-Rius & Castillo, 2016 [20] | 4 | Archidona-Yuste et al. [20] |
| 1. *Xiphinema paradentatum* | Barsi, Fanelli & De Luca, 2017 [42] | 5 | Barsi et al. [42] |
| 1. *Xiphinema pirinense* | Mincheva, Lazarova & Peneva, 2008 [43] | 4 | Mincheva et al. [43] |
| 1. *Xiphinema poasense* | Varela-Benavides, Peraza-Padilla, Cantalapiedra-Navarrete, Palomares-Rius, Castillo, & Archidona-Yuste, 2018 [44] | 2 | Varela-Benavides et al. [44] |
| 1. *Xiphinema pombalense* | Bravo & Lamberti, 1996 [45] | 8 | Bravo & Lamberti [45] |
| 1. *Xiphinema pseudokrugi* | Swart & Quénéhervé, 1998 [34] | 2 | Swart & Quénéhervé [34] |
| 1. *Xiphinema robbinsi* | Pedram, Niknam & Decraemer, 2008 [46] | 6 | Pedram et al. [46] |
| 1. *Xiphinema savaryi* | Lamberti, Troccoli & Agostinelli, 1997 [47] | 7 | Lamberti et al. [47] |
| 1. *Xiphinema seinhorsti* | Swart & Quénéhervé, 1998 [34] | 5 | Swart & Quénéhervé [34] |
| 1. *Xiphinema sharonae* | Malan, Swart, Meyer & Heyns, 1997 [48] | 5 | Malan et al. [48] |
| 1. *Xiphinema siamense* | Lamberti, Troccoli & Agostinelli, 1997 [47] | 1 | Lamberti et al. [47] |
| 1. *Xiphinema silvesi* | Roca & Bravo, 1998 [49] | 5 | Roca & Bravo [49] |
| 1. *Xiphinema simpliciforme* | Coomans & Heyns, 1997 [39] | 5 | Coomans & Heyns [39] |
| 1. *Xiphinema souchaudi* | Baujard, Luc & Reversat, 1998 [50] | 5 | Baujard et al. [50] |
| 1. *Xiphinema sphaerocephalum* (R) | Lamberti, Castillo, Gomez-Barcina & Agostinelli, 1992 [18] | 6 | Gutiérrez‐Gutiérrez et al. [19] |
| 1. *Xiphinema tica* | Peraza-Padilla, Cantalapiedra-Navarrete, Zamora-Araya, Palomares-Rius, Castillo, Archidona-Yuste, 2017 [51] | 4 | Peraza-Padilla et al. [51] |
| 1. *Xiphinema torvum* | Siddiqi, 2000 [26] | 2 | Siddiqi [26] |
| 1. *Xiphinema turdetanensis* | Gutiérrez‐Gutiérrez, Cantalapiedra-Navarrete, Remesal, Palomares-Rius, Navas-Cortes & Castillo, 2013 [21] | 5 | Gutiérrez‐Gutiérrez et al. [21] |
| 1. *Xiphinema variegatum* | Siddiqi, 2000 [26] | 2 | Siddiqi [26] |
| 1. *Xiphinema variurum* | Barsi & Lamberti, 1998 [52] | 8 | Barsi & Lamberti [52] |
| 1. *Xiphinema vicarium* | Siddiqi, 2000 [26] | 2 | Siddiqi [26] |
| 1. *Xiphinema winotoi* | Razak & Loof, 1998 [53] | 1 | Razak & Loof [53] |
| 1. *Xiphinema zagrosense* | Ghaemi, Pourjam, Pedram, Robbins, Ye & Decraemer, 2012 [54] | 6 | Ghaemi et al. [54] |
| 1. *Xiphinema zyzy* | Heyns & Swart, 2002 [55] | 5 | Heyns &Swart [55] |

R: Revalidated in the given reference

*: Might not belongs to *Xiphinema*, by having small amphidial slit (needs molecular study in future, for further confirmations).
