# Supplementary material for: Genetic diversity, and description of a new dagger nematode, Xiphinema afratakhtehnsis sp. nov., (Dorylaimida: Longidoridae) in natural forests of southeastern Gorgan, northern Iran
Source: PLoS One. 2019 May 1;14(5):e0214147. doi: 10.1371/journal.pone.0214147 (PMC6493718; doi:10.1371/journal.pone.0214147)
Supplement: S2 Table — (DOCX) [file pone.0214147.s003.docx]

**S2 Table**.

| **Isolate (code)** | **Sequenced locus** | **Accession number** | **GPS info.** | **Province, locality** | **Host plant** |
| --- | --- | --- | --- | --- | --- |
| 801A | 18S | MH429072 | N 36˚47.990’, E 54˚56.056’ | Golestan, Afrātakhteh forests | *Pteridium aquilinum* |
| 801B | D2-D3 | MH429073 | N 36˚47.990’, E 54˚56.056’ | Golestan, Afrātakhteh forests | *Pteridium aquilinum* |
| 801E | D2-D3 | MH429074 | N 36˚47.990’, E 54˚56.056’ | Golestan, Afrātakhteh forests | *Pteridium aquilinum* |
| 801E1 | D2-D3 | MH429075 | N 36˚47.990’, E 54˚56.056’ | Golestan, Afrātakhteh forests | *Pteridium aquilinum* |
| 801F | D2-D3 | MH429076 | N 36˚47.990’, E 54˚56.056’ | Golestan, Afrātakhteh forests | *Pteridium aquilinum* |
| 803K | D2-D3 | MH429077 | N 36˚47.718’, E 54˚55.028’ | Golestan, Afrātakhteh forests | *Pteridium aquilinum* |
| 803K1 | D2-D3 | MH429078 | N 36˚47.718’, E 54˚55.028’ | Golestan, Afrātakhteh forests | *Pteridium aquilinum* |
| 764M | D2-D3 | MH429080 | N 36˚46.863’, E 54˚27.080’ | Golestan, Alangdareh forests | *Rubus* sp. |
| Not assigned | D2-D3 | MH429079 | ? | ? | ? |
| 764M1 | D2-D3 | MH429081 | N 36˚46.863’, E 54˚27.080’ | Golestan, Alangdareh forests | *Rubus* sp. |
| 762S | D2-D3 | MH429082 | N 36˚40.460’, E 54˚27.858’ | Golestan, forests close to Abshar road | *Rubus* sp. |
| 762S1 | D2-D3 | MH429083 | N 36˚40.460’, E 54˚27.858’ | Golestan, forests close to Abshar road | *Rubus* sp. |
| 779ab | D2-D3 | MH429084 | N 36˚50.038’, E 54˚42.896’ | Golestan, Mohammad Abad forests | *Pteridium aquilinum* |
| 779ab1 | D2-D3 | MH429085 | N 36˚50.038’, E 54˚42.896’ | Golestan, Mohammad Abad forests | *Pteridium aquilinum* |
| 801C | ITS1 | MH429086 | N 36˚47.990’, E 54˚56.056’ | Golestan, Afrātakhteh forests | *Pteridium aquilinum* |
| 801C1 | ITS1 | MH429087 | N 36˚47.990’, E 54˚56.056’ | Golestan, Afrātakhteh forests | *Pteridium aquilinum* |
| 801I | ITS1 | MH429088 | N 36˚47.990’, E 54˚56.056’ | Golestan, Afrātakhteh forests | *Pteridium aquilinum* |
| 801I1 | ITS1 | MH429089 | N 36˚47.990’, E 54˚56.056’ | Golestan, Afrātakhteh forests | *Pteridium aquilinum* |
| 801J | ITS1 | MH429090 | N 36˚47.990’, E 54˚56.056’ | Golestan, Afrātakhteh forests | *Pteridium aquilinum* |
| 801J1 | ITS1 | MH429091 | N 36˚47.990’, E 54˚56.056’ | Golestan, Afrātakhteh forests | *Pteridium aquilinum* |
| 764N | ITS1 | MH429092 | N 36˚46.863’, E 54˚27.080’ | Golestan, Alangdareh forests | *Rubus* sp. |
| 764N1 | ITS1 | MH429093 | N 36˚46.863’, E 54˚27.080’ | Golestan, Alangdareh forests | *Rubus* sp. |
| 762T | ITS1 | MH429094 | N 36˚40.460’, E 54˚27.858’ | Golestan, forests close to Abshar road | *Rubus* sp. |
| 762T1 | ITS1 | MH429095 | N 36˚40.460’, E 54˚27.858’ | Golestan, forests close to Abshar road | *Rubus* sp. |
| 405X | ITS1 | MH429096 | N 36˚45.976’, E 55˚01.282’ | Semnan province, Clouds forest | *Quercus* sp. |
| 405X1 | ITS1 | MH429097 | N 36˚45.976’, E 55˚01.282’ | Semnan province, Clouds forest | *Quercus* sp. |
| 801D | COI | MH429098 | N 36˚47.990’, E 54˚56.056’ | Golestan, Afrātakhteh forests | *Pteridium aquilinum* |
| 801D1 | COI | MH429099 | N 36˚47.990’, E 54˚56.056’ | Golestan, Afrātakhteh forests | *Pteridium aquilinum* |
| 801G | COI | MH429100 | N 36˚47.990’, E 54˚56.056’ | Golestan, Afrātakhteh forests | *Pteridium aquilinum* |
| 801G1 | COI | MH429101 | N 36˚47.990’, E 54˚56.056’ | Golestan, Afrātakhteh forests | *Pteridium aquilinum* |
| 801H | COI | MH429102 | N 36˚47.990’, E 54˚56.056’ | Golestan, Afrātakhteh forests | *Pteridium aquilinum* |
| 801H1 | COI | MH429103 | N 36˚47.990’, E 54˚56.056’ | Golestan, Afrātakhteh forests | *Pteridium aquilinum* |
| 764P | COI | MH429104 | N 36˚46.863’, E 54˚27.080’ | Golestan, Alangdareh forests | *Rubus* sp. |
| 764P1 | COI | MH429105 | N 36˚46.863’, E 54˚27.080’ | Golestan, Alangdareh forests | *Rubus* sp. |
| 764Q | COI | MH429106 | N 36˚46.863’, E 54˚27.080’ | Golestan, Alangdareh forests | *Rubus* sp. |
| 764Q1 | COI | MH429107 | N 36˚46.863’, E 54˚27.080’ | Golestan, Alangdareh forests | *Rubus* sp. |
| 805W | COI | MH429108 | N 36˚48.129’, E 54˚58.205’ | Golestan, Afrātakhteh forests | *Pteridium aquilinum* |
| 805W1 | COI | MH429109 | N 36˚48.129’, E 54˚58.205’ | Golestan, Afrātakhteh forests | *Pteridium aquilinum* |
| 790Y | COI | MH429110 | N 36˚52.865’, E 54˚53.288’ | Golestan, close to Kaboudwal waterfall | *Hedera helix* |
| 790Y1 | COI | MH429111 | N 36˚52.865’, E 54˚53.289’ | Golestan, close to Kaboudwal waterfall | *Hedera helix* |
| 779Z | COI | MH429112 | N 36˚50.038’, E 54˚42.896’ | Golestan, Mohammad Abad forests | *Pteridium aquilinum* |
| 779Z1 | COI | MH429113 | N 36˚50.038’, E 54˚42.896’ | Golestan, Mohammad Abad forests | *Pteridium aquilinum* |
| 770 | - | - | N 36 ˚46.150’, E54 ˚34.568’ | Golestan, Touskestan forests | *Quercus* sp. |
| 775 | - | - | N 36 ˚45.648’, E54 ˚35.023’ | Golestan, Touskestan forests | *Quercus* sp. |
| 806 | - | - | N 36 ˚48.703’, E54 ˚59.825’ | Golestan, Afrātakhteh forests | *Quercus* sp. |
| 792 | - | - | N 36 ˚51.860’, E54 ˚57.432’ | Zarringol, Shirinābād forests | *Quercus* sp. |
| 773 | - | - | N 36 ˚45.351’, E54 ˚34.932’ | Golestan, Touskestan forests | *Quercus* sp. |
| 787 | - | - | N 36 ˚48.623’, E54 ˚51.086’ | Golestan, Touskestan forests | *Quercus* sp. |
| 794 | - | - | N 36 ˚49.154’, E54 ˚59.602’ | Golestan, Afrātakhteh forests | *Quercus* sp. |
| 797 | - | - | N 36 ˚47.895’, E54 ˚58.125’ | Golestan, Afrātakhteh forests | *Quercus* sp. |
| 799 | - | - | N 36 ˚47.706’, E54 ˚57.277’ | Golestan, Afrātakhteh forests | *Quercus* sp. |
| A2 | - | - | N 36 ˚46.506’, E54 ˚26.605’ | Golestan, Alangdareh forests | *Quercus* sp. |
| 798 | - | - | N 36 ˚47.993’, E54 ˚57.763’ | Golestan, Afrātakhteh forests | *Quercus* sp. |
| 796 | - | - | N 36 ˚47.878’, E54 ˚58.081’ | Golestan, Afrātakhteh forests | *Quercus* sp. |
| 804 | - | - | N 36 ˚47.808’, E54 ˚55.138’ | Golestan, Afrātakhteh forests | *Quercus* sp. |
| 783 | - | - | N 36 ˚48.221’, E54 ˚49.694’ | Golestan, close to Rig Cheshmeh | *Quercus* sp. |
| 784 | - | - | N 36 ˚48.439’, E54 ˚50.630’ | Golestan, close to Rig Cheshmeh | *Quercus* sp. |
| A1 | ? | ? | ? | Golestan province | ? |

(-) Not obtained or not performed.

(?) Missing data
