# Supplementary material for: Genetic diversity, and description of a new dagger nematode, Xiphinema afratakhtehnsis sp. nov., (Dorylaimida: Longidoridae) in natural forests of southeastern Gorgan, northern Iran
Source: PLoS One. 2019 May 1;14(5):e0214147. doi: 10.1371/journal.pone.0214147 (PMC6493718; doi:10.1371/journal.pone.0214147)
Supplement: S3 Table — (DOCX) [file pone.0214147.s004.docx]

**S3 Table .**

| **Code** | **Female** | | | | | | | | | | | | | **Male** | **Females** |
| --- | --- | --- | --- | --- | --- | --- | --- | --- | --- | --- | --- | --- | --- | --- | --- |
| **Character/Ratio**^b^ | **770** | **773** | **775** | **790** | **794** | **804** | **806** | **A1** | **A2** | **797** | **779** | **787** | **805** | **805** | **Total range** |
| n | 1 | 1 | 1 | 1 | 1 | 1 | 1 | 1 | 1 | 2 | 4 | 10 | 10 | 1 | 74 |
| L | 4142.5 | 3972.5 | 3743.8 | 3721.3 | 4365 | 3507.5 | 4163.8 | 3590 | 3315 | 3950.6±195.3 | 4111.3±134.6 | 3721.3±182.2 | 3991.4±92.8 | 4140 | 4029.5±289.1 |
|  |  |  |  |  |  |  |  |  |  | (3813-4089) | (3920-4225) | (3418-3935) | (3750-4080) |  | (3315-4885) |
| a | 56.7 | 52.3 | 57.6 | 57.3 | 58.2 | 51.6 | 53.4 | 52 | 48 | 57.4±5.2 | 57.1±4.6 | 57.9±3.1 | 57.3±2.5 | 71.4 | 58.6±5.6 |
|  |  |  |  |  |  |  |  |  |  | (53.7-61.0) | (52.3-62.1) | (53.6-62.1) | (54.1-62.8) |  | (48.0-74.3) |
| b | 6.5 | 5.3 | 6.7 | 5.9 | 6.7 | 5.4 | 6.6 | 6.8 | 5.8 | 6.1±0.2 | 6.4±0.2 | 6.1±0.2 | 6.6±0.2 | 6.4 | 6.5±0.5 |
|  |  |  |  |  |  |  |  |  |  | (5.9-6.3) | (6.1-6.6) | (5.8-6.5) | (6.2-6.8) |  | (5.3-8.5) |
| c | 84.5 | 88.3 | 87.1 | 87.6 | 79.4 | 81.6 | 77.1 | 74.8 | 75.3 | 85.4±10.7 | 86.1±2.6 | 88.3±5.7 | 87.0±6.7 | 75.3 | 88.0±7.6 |
|  |  |  |  |  |  |  |  |  |  | (77.8-92.9) | (83.4-88.9) | (77.7-96.0) | (77.1-95.8) |  | (74.5-106.9) |
| c' | 0.9 | 0.8 | 0.8 | 1 | 0.9 | 0.8 | 0.9 | 0.9 | 0.8 | 0.8±0.1 | 0.8±0.1 | 0.8±0.01 | 0.9±0.001 | 1.1 | 0.8±0.1 |
|  |  |  |  |  |  |  |  |  |  | (0.8-0.9) | (0.8-0.9) | (0.7-0.9) | (0.8-0.9) |  | (0.7-1.1) |
| V | 49.1 | 53.3 | 52.1 | 50.8 | 51.9 | 52.6 | 50.4 | 51.2 | 53.6 | 53.0±0.1 | 50.6±0.7 | 51.3±1.8 | 49.8±1.6 | - | 51.0±2.1 |
|  |  |  |  |  |  |  |  |  |  | (53.0-53.1) | (49.7-51.4) | (47.4-53.6) | (48.2-53.4) |  | (47.2-58.5) |
| Lip height | 8 | 8 | 8 | 10 | 9 | 8 | 8 | 8 | 10 | 9.5±0.7 | 8.3±0.5 | 9.1±0.9 | 8.9±0.8 | 10 | 8.9±0.8 |
|  |  |  |  |  |  |  |  |  |  | (9-10) | (8-9) | (8-10) | (8-10) |  | (8-10) |
| Lip width | 16 | 16 | 16 | 18 | 17 | 16 | 16 | 16 | 18 | 17.5±0.7 | 16.3±0.5 | 17.1±0.9 | 16.9±0.9 | 19 | 16.8±0.8 |
|  |  |  |  |  |  |  |  |  |  | (17-18) | (16-17) | (16-18) | (16-18) |  | (16-18) |
| Odontostyle length | 162 | 164 | 156 | 160 | 172 | 171 | 165 | 155 | 155 | 166.0±4.2 | 171.1±1.9 | 160.9±2.3 | 161.0±2.4 | 165 | 164.3±4.9 |
|  |  |  |  |  |  |  |  |  |  | (163-169) | (169-173) | (157-163) | (158-164) |  | (155-173) |
| Odontophore length | 97 | 100 | 93 | 91 | 97 | 98 | 102 | 99 | 99 | 100.0±2.8 | 97.3±3.2 | 98.0±2.6 | 97.1±2.7 | 99 | 98.4±4.0 |
|  |  |  |  |  |  |  |  |  |  | (98-102) | (95-102) | (93-101) | (92-101) |  | (89-107) |
| Stylet total length | 259 | 264 | 249 | 251 | 269 | 269 | 267 | 252 | 254 | 266.0±7.1 | 268.2±2.6 | 258.7±4.7 | 258.1±3.6 | 264 | 261.9±9.0 |
|  |  |  |  |  |  |  |  |  |  | (261-271) | (266-271) | (248-264) | (250-263) |  | (223-277) |
| Replacement odontostyle | - | - | - | - | - | - | - | - | - | - | - | - | - | - | - |
| Anterior end to vulva | 2035 | 2117.5 | 1950 | 1888.8 | 2265 | 1845 | 2100 | 1837.5 | 1777.5 | 2095.0±106.1 | 2081.9±92.1 | 1906.5±77.6 | 1987.8±68.9 | - | 2052.4±141.1 |
|  |  |  |  |  |  |  |  |  |  | (2020-2170) | (1950-2150) | (1775-2035) | (1922.5-2147.5) |  | (1775.0-2456.3) |
| Anterior end to guiding ring | 151 | 146 | 137 | 157 | 150 | 157 | 155 | 144 | 140 | 148.5±2.1 | 156.8±2.4 | 147.3±6.0 | 148.8±4.5 | 154 | 152.7±9.2 |
|  |  |  |  |  |  |  |  |  |  | (147-150) | (155-160) | (140-155) | (143-159) |  | (135-176) |
| Flange width | - | - | - |  | - | - | - | - | - | - | - | - | - | 15 | 14.5±1.7 |
|  |  |  |  |  |  |  |  |  |  |  |  |  |  |  | (12-19) |
| Pharynx length | 632.5 | 747.5 | 560 | 632.5 | 652.5 | 650 | 632.5 | 530 | 572.5 | 647.5±7.1 | 644.4±16.1 | 606.5±24.1 | 608.3±11.8 | 645 | 624.5±41.2 |
|  |  |  |  |  |  |  |  |  |  | (642.5-652.5) | (630.0-667.5) | (557.5-650.0) | (595-630) |  | (530.0-747.5) |
| Pharyngeal expansion length | 147 | 154 | 134 | 136 | 144 | 153 | 152 | 131 | 134.5 | 149.8±8.1 | 148.9±5.2 | 142.7±11.0 | 143.4±8.2 | 148 | 145.0±10.2 |
|  |  |  |  |  |  |  |  |  |  | (144.0-155.5) | (145.0-156.5) | (123-156) | (135-164) |  | (122-165) |
| Pharyngeal expansion diam. | 30 | 29 | 28 | 28 | 32 | 28 | 32 | 29 | 31 | 28.5±0.7 | 29.8±1.7 | 28.5±1.4 | 28.9±1.0 | 29 | 30.4±2.7 |
|  |  |  |  |  |  |  |  |  |  | (28-29) | (28-32) | (27-31) | (27-30) |  | (27-37) |
| Body width at mid body | 73 | 76 | 65 | 65 | 75 | 68 | 78 | 69 | 69 | 69.0±2.8 | 72.3±3.9 | 64.5±6.0 | 69.7±2.6 | 58 | 69.2±6.0 |
|  |  |  |  |  |  |  |  |  |  | (67-71) | (68-76) | (55-71) | (65-74) |  | (55-79) |
| - at anus | 56 | 59 | 51 | 44 | 61 | 51 | 63 | 55 | 58 | 55.5±0.7 | 60.0±2.7 | 50.4±2.9 | 52.5±2.4 | 52 | 54.6±5.0 |
|  |  |  |  |  |  |  |  |  |  | (55-56) | (56-62) | (46-54) | (48-57) |  | (44-67) |
| - at guiding ring level | 50 | 49 | 44 | 43 | 49 | 47 | 56 | 45 | 54 | 62.5±6.4 | 51.3±3.4 | 47.6±4.5 | 49.5±2.0 | 46 | 50.1±5.0 |
|  |  |  |  |  |  |  |  |  |  | (58-67) | (48-56) | (44-56) | (46-52) |  | (41-67) |
| Prerectum length | 730 | 975 | 577.5 | 487.5 | 832 | 440 | 777.5 | 352.5 | 550 | 536.3±23.0 | 525.6±33.6 | 456.7±51.1 | 522.3±59.0 | - | 568.2±114.2 |
|  |  |  |  |  |  |  |  |  |  | (520.0-552.5) | (482.5-560.0) | (397.5-545.0) | (432.5-665.0) |  | (352.5-975.0) |
| Rectum length | 62.5 | 47 | 47.5 | 50 | 47.5 | 37.5 | 41.3 | 50 | 35 | 45.0±7.1 | 50±2 | 44.0±3.8 | 44.3±6.6 | - | 48.0±6.7 |
|  |  |  |  |  |  |  |  |  |  | (40-50) | (47.5-52.5) | (40.0-52.5) | (37.5-57.5) |  | (35.0-65.5) |
| Hyaline tail region | 14 | 19 | 19 | 18 | 18 | 14 | 15 | 15 | 16 | 12.5±3.5 | 15.8±1.0 | 15.4±1.3 | 16.6±1.4 | 13 | 15.3±1.7 |
|  |  |  |  |  |  |  |  |  |  | (10-15) | (15-17) | (13-18) | (15-19) |  | (10-19) |
| Tail | 49 | 45 | 43 | 42.5 | 55 | 43 | 54 | 48 | 44 | 46.5±3.5 | 47.8±1.5 | 42.2±1.5 | 46.1±3.4 | 55 | 46.0±4.3 |
|  |  |  |  |  |  |  |  |  |  | (44-49) | (47-50) | (40-45) | (42-51) |  | (38-61) |
| Spicules | - | - | - | - | - | - | - | - | - | - | - | - | - | 83 | - |

(-) Not obtained or not performed.

^a^ Measurements are in µm and in the form: mean ± standard deviation (range).

^b^ Abbreviations as defined in Jairajpuri & Ahmad [62]. a, body length/maximum body width; b, body length/pharyngeal length; c, body length/tail length; c', tail length/body width at anus; V (distance from anterior end to vulva/body length) x 100.
