# Supplementary material for: Genetic diversity, and description of a new dagger nematode, Xiphinema afratakhtehnsis sp. nov., (Dorylaimida: Longidoridae) in natural forests of southeastern Gorgan, northern Iran
Source: PLoS One. 2019 May 1;14(5):e0214147. doi: 10.1371/journal.pone.0214147 (PMC6493718; doi:10.1371/journal.pone.0214147)
Supplement: S4 Table — (DOCX) [file pone.0214147.s005.docx]

**S4 Table.**

| **Accession numbers** | **1** | **2** | **3** | **4** | **5** | **6** | **7** | **8** | **9** | **10** | **11** | **12** | **13** | **14** | **15** | **16** |
| --- | --- | --- | --- | --- | --- | --- | --- | --- | --- | --- | --- | --- | --- | --- | --- | --- |
| 1. **MH429098** |  |  |  |  |  |  |  |  |  |  |  |  |  |  |  |  |
| 1. **MH429099** | 0.0 |  |  |  |  |  |  |  |  |  |  |  |  |  |  |  |
| 1. **MH429100** | 0.0 | 0.0 |  |  |  |  |  |  |  |  |  |  |  |  |  |  |
| 1. **MH429101** | 0.0 | 0.0 | 0.0 |  |  |  |  |  |  |  |  |  |  |  |  |  |
| 1. **MH429102** | 0.0 | 0.0 | 0.0 | 0. |  |  |  |  |  |  |  |  |  |  |  |  |
| 1. **MH429103** | 0.0 | 0.0 | 0.0 | 0.0 | 0.0 |  |  |  |  |  |  |  |  |  |  |  |
| 1. **MH429104** | 13.6 | 13.6 | 13.6 | 13.6 | 13.6 | 13.6 |  |  |  |  |  |  |  |  |  |  |
| 1. **MH429105** | 13.6 | 13.6 | 13.6 | 13.6 | 13.6 | 13.6 | 0.0 |  |  |  |  |  |  |  |  |  |
| 1. **MH429106** | 13.6 | 13.6 | 13.6 | 13.6 | 13.6 | 13.6 | 0.0 | 0.0 |  |  |  |  |  |  |  |  |
| 1. **MH429107** | 13.6 | 13.6 | 13.6 | 13.6 | 13.6 | 13.6 | 0.0 | 0.0 | 0.0 |  |  |  |  |  |  |  |
| 1. **MH429110** | 10.6 | 10.6 | 10.6 | 10.6 | 10.6 | 10.6 | 14.1 | 14.1 | 14.1 | 14.1 |  |  |  |  |  |  |
| 1. **MH429111** | 10.6 | 10.6 | 10.6 | 10.6 | 10.6 | 10.6 | 14.1 | 14.1 | 14.1 | 14.1 | 0.0 |  |  |  |  |  |
| 1. **MH429112** | 12.7 | 12.7 | 12.7 | 12.7 | 12.7 | 12.7 | 16.3 | 16.3 | 16.3 | 16.3 | 14.0 | 14.0 |  |  |  |  |
| 1. **MH429113** | 12.7 | 12.7 | 12.7 | 12.7 | 12.7 | 12.7 | 16.3 | 16.3 | 16.3 | 16.3 | 14.0 | 14.0 | 0.0 |  |  |  |
| 1. **MH429108** | 0.0 | 0.0 | 0.0 | 0.0 | 0.0 | 0.0 | 13.6 | 13.6 | 13.6 | 13.6 | 10.6 | 10.6 | 12.7 | 12.7 |  |  |
| 1. **MH429109** | 0.0 | 0.0 | 0.0 | 0.0 | 0.0 | 0.0 | 13.6 | 13.6 | 13.6 | 13.6 | 10.6 | 10.6 | 12.7 | 12.7 | 0.0 |  |

^a^ values in percentage.
