# Supplementary material for: Genetic diversity, and description of a new dagger nematode, Xiphinema afratakhtehnsis sp. nov., (Dorylaimida: Longidoridae) in natural forests of southeastern Gorgan, northern Iran
Source: PLoS One. 2019 May 1;14(5):e0214147. doi: 10.1371/journal.pone.0214147 (PMC6493718; doi:10.1371/journal.pone.0214147)
Supplement: S5 Table — (DOCX) [file pone.0214147.s006.docx]

**S5 Table*.***

| Haplotype code | Accession number, species , isolate code |
| --- | --- |
| Hap1 | MH429098 *Xiphinema afratakhtehnsis* sp. nov. Isolate 801D |
| Hap1 | MH429099 *Xiphinema afratakhtehnsis* sp. nov. Isolate 801D1 |
| Hap1 | MH429100 *Xiphinema afratakhtehnsis* sp. nov. Isolate 801G |
| Hap1 | MH429101 *Xiphinema afratakhtehnsis* sp. nov. Isolate 801G1 |
| Hap1 | MH429102 *Xiphinema afratakhtehnsis* sp. nov. Isolate 801H |
| Hap1 | MH429103 *Xiphinema afratakhtehnsis* sp. nov. Isolate 801H1 |
| Hap1 | MH429108 *Xiphinema afratakhtehnsis* sp. nov. Isolate 805W |
| Hap1 | MH429109 *Xiphinema afratakhtehnsis* sp. nov. Isolate 805W1 |
| Hap2 | MH429104 *Xiphinema afratakhtehnsis* sp. nov. Isolate 764P |
| Hap2 | MH429105 *Xiphinema afratakhtehnsis* sp. nov. Isolate 764P1 |
| Hap2 | MH429106 *Xiphinema afratakhtehnsis* sp. nov. Isolate 764Q |
| Hap2 | MH429107 *Xiphinema afratakhtehnsis* sp. nov. Isolate 764Q1 |
| Hap3 | MH429110 *Xiphinema afratakhtehnsis* sp. nov. Isolate 790Y |
| Hap3 | MH429111 *Xiphinema afratakhtehnsis* sp. nov. Isolate 790Y1 |
| Hap4 | MH429112 *Xiphinema afratakhtehnsis* sp. nov. Isolate 779Z |
| Hap4 | MH429113 *Xiphinema afratakhtehnsis* sp. nov. Isolate 779Z1 |
